# Heart rate variability analysis in comorbid insomnia and sleep apnea (COMISA)

**DOI:** 10.1038/s41598-025-02541-7

**Published:** 2025-05-21

**Authors:** Adrián Martín-Montero, Fernando Vaquerizo-Villar, Clara García-Vicente, Gonzalo C. Gutiérrez-Tobal, Thomas Penzel, Roberto Hornero

**Affiliations:** 1 Biomedical Engineering Group, University of Valladolid, Av. Ramón y Cajal, 7, Valladolid, 47003 Spain; 2 Centro de Investigación Biomédica en Red en Bioingeniería, Biomateriales y Nanomedicina, CIBER-BBN, Valladolid, Spain; 3 Interdisciplinary Center of Sleep Medicine, Charité-Universitätsmedizin, Berlin, Germany

**Keywords:** Autonomic nervous system (ANS), Comorbid insomnia and sleep apnea (COMISA), Heart rate variability (HRV), Insomnia, Obstructive sleep apnea (OSA), Biomarkers, Comorbidities, Biomedical engineering

## Abstract

Obstructive sleep apnea (OSA) and insomnia are the two most prevalent sleep disorders, often co-occurring in a condition termed comorbid insomnia and sleep apnea (COMISA). While autonomic nervous system (ANS) dysfunction resulting from each of these disorders has been separately established through heart rate variability (HRV) analysis, the specific overnight ANS alterations due to COMISA have not been explored. This study aims to characterize nocturnal ANS alterations attributable to COMISA through time and frequency HRV analysis, distinguishing them from those of isolated insomnia or OSA. A total of 5,335 electrocardiograms from the Sleep Heart Health Study (SHHS) dataset were included in this research. Based on overnight polysomnography and sleep questionnaires, participants were categorized into No-OSA (2,738 subjects), Insomnia (190 subjects), OSA (2,260 subjects), or COMISA (147 subjects) groups. Classic time and frequency HRV features, along with specific frequency measures, were computed to characterize HRV behavior throughout the whole night, in both wakefulness and sleep periods. The analysis revealed that COMISA-specific ANS dysfunction manifests as reduced parasympathetic activity during wakefulness and heightened sympathetic activation during sleep. While primary ANS dysfunctions seem to result from recurrent apneic events affecting frequency features present in both OSA and COMISA, insomnia significantly alters mean heart rate during sleep, thus being the only feature distinguishing these two conditions. In conclusion, the combined effects of OSA and insomnia induce specific ANS dysfunction at night, highlighting the need for further HRV studies to better understand the impact of COMISA on ANS and its cardiovascular implications.

*Clinical trial registration*: The clinical trial identifier of the original SHHS database is NCT00005275, https://sleepdata.org/datasets/shhs.

**Supplementary Information:**

The online version contains supplementary material available at 10.1038/s41598-025-02541-7.

## Introduction

The underlying physiological mechanisms that take place during sleep are fundamental to maintain health throughout life^1^. Restful sleep is vital for brain health and the proper functioning of essential processes such as metabolism, immune response, hormone regulation, and cardiovascular systems^1–3^. Key indicators of healthy sleep include enough sleep duration, high sleep quality, and a sleep routine regarding timing and regularity^1,2^. However, sleep disorders disrupt these factors, thereby posing a significant health risk to affected individuals^3,4^. Among the recognized sleep disorders, the two most prevalent are obstructive sleep apnea (OSA) and insomnia, affecting between 10 and 30% of the general adult population^5^.

Insomnia symptoms are characterized by self-reported difficulties related to nocturnal sleep disturbances, including troubles initiating sleep, maintaining sleep, or experiencing early awakenings without being able to resume sleep^6^. These symptoms can even occur in combination on the same night for the same patient^6^. A diagnosis of insomnia is confirmed when these nocturnal symptoms appear repeatedly, cause daytime impairments, and last for more than three months^6^. On the other hand, OSA is a sleep-disordered breathing condition defined by repetitive partial or complete pauses in respiration (hypopneas and apneas, respectively) during sleep^7^. The diagnosis of OSA is based on overnight polysomnography (PSG), a procedure in which various biological signals are recorded and subsequently analyzed by medical experts to determine the apnea-hypopnea index (AHI). This index quantifies the number of apneic events per hour of sleep (e/h), thus indicating both the presence and severity of the disease^7,8^.

By definition, OSA events occur during sleep, while insomnia is characterized by sleep deprivation, resulting in both diseases sometimes being considered as opposite conditions. However, these disorders often coexist in the same patients^9^. Studies show that 30–40% of individuals diagnosed with insomnia meet the criteria for OSA, and up to half of those with OSA report insomnia symptoms^5,10–12^. The first study to report the coexistence of OSA and insomnia was conducted by Guilleminault et al. in 1973 ^13^. Despite this early recognition, research on this comorbidity remained limited for several decades^9^. Interest in the topic resurged in 2017 when Sweetman et al. coined the term “co-morbid insomnia and obstructive sleep apnea” (COMISA)^9^. Thus, COMISA is considered a relatively emerging field of research.

Both insomnia and OSA have been independently associated with cardiovascular diseases, such as hypertension and congestive heart failure^14–16^. Autonomic dysfunction has been identified as a key physiological mechanism contributing to the increased risk of these conditions^14,16^. In this regard, autonomic nervous system (ANS) affections have been extensively studied through heart rate variability (HRV) analysis in both OSA^15,17–20^ and insomnia^16,21,22^. For OSA, the common finding is that autonomic dysfunction emerges by heightened sympathetic nervous system (SNS) activity during apneic events^14,18,19^. For insomnia, evidence suggests that ANS dysfunction also arises from SNS activation due to sleep deficiency and a state of hyper-arousal^16,21,22^. The pathophysiological impacts of insomnia and OSA are combined in COMISA patients^23^. Consequently, COMISA has been linked to poorer sleep and life quality, impaired daytime functioning, increased all-cause mortality risk, and a higher likelihood of developing cardiovascular diseases compared to either condition alone^23–25^. Regarding ANS alterations, only a very recent work has analyzed the COMISA impact on pre-sleep HRV measures^26^. However, the combined effect of both diseases on overnight autonomic dysfunction has not yet been evaluated.

Based on these considerations, we hypothesize that the coexistence of insomnia and OSA leads to a distinct fingerprint on the ANS behavior of COMISA patients, which can be characterized through overnight HRV analysis. Consequently, the primary aim of this study is to characterize specific nocturnal ANS alterations attributable to COMISA through time and frequency HRV analysis, enabling differentiation of its combined effects from those of insomnia or OSA alone. Therefore, the noteworthy contributions of this study are: (i) first analysis of HRV activity during nighttime, distinguishing between wakefulness and sleep, and highlighting the combined impact of insomnia and OSA on ANS dysfunction within the same patient; (ii) the adaptation and evaluation of OSA-specific frequency features, previously identified in pediatric populations, for use in adults with OSA and COMISA; and (iii) the introduction of a novel COMISA-specific HRV spectral feature, proposed here for the first time.

## Methods

### Subjects and signals

The present study analyzed 5,335 nocturnal PSGs from adults aged over 40 years, sourced from the Sleep Heart Health Study (SHHS) database (Clinical Trial Number: NCT00005275). The SHHS study was carried out according to the Declaration of Helsinki. In all patients, written information was provided, and the study was approved by the ethical review board at each site (https://sleepdata.org/datasets/shhs/files/m/browser/documentation/SHHS1_Protocol.pdf?inline=1). SHHS was a multicenter cohort study designed to evaluate cardiovascular and other consequences of sleep-disordered breathing. Detailed descriptions of the study design and methodology have been previously reported^27^, and data are publicly accessible upon request at https://sleepdata.org/datasets/shhs. The original SHHS dataset comprises 5,804 subjects who underwent a baseline clinic visit between November 1995 and January 1998. From those 5,804 initial subjects, 469 participants were excluded due to missing information on insomnia symptoms or issues encountered during the processing stage (details provided below). This resulted in the final sample of 5,335 subjects. At the time of the clinic visit, patients underwent overnight PSG, during which electrocardiogram (ECG) signals were recorded at frequencies of 125–250 Hz. Sleep studies were annotated by medical sleep specialists following a strict standardized procedure^28^, which subsequently allowed for the extraction of the AHI.

At the time the original SHHS study was conducted, a standardized measure of insomnia was not included. Nevertheless, aligning with previous works that used the SHHS dataset to investigate COMISA^24,25,29^, the definition of Insomnia, COMISA, and OSA groups can be established based on current insomnia and COMISA diagnostic criteria^9^. In addition to the PSG studies, participants completed questionnaires regarding insomnia symptoms, sleep habits, and daytime functioning. Based on their answers, insomnia criteria were defined as self-reporting at least one nocturnal symptom more than 16 times per month, accompanied by daytime impairment^24,25,29^. Nocturnal symptoms included difficulties initiating sleep, maintaining sleep, and/or waking up too early and being unable to resume sleep. Daytime impairments were characterized as feeling unrested for more than 5 days per month, feeling tired most of the time, and/or having little to no energy over the past month. OSA was defined as subjects with an AHI ≥ 15 e/h^24,25,29^. Thus, a subject who met both insomnia and OSA criteria was classified as having COMISA (147 subjects). Subjects meeting only the insomnia criteria or the OSA criteria were placed in the Insomnia (190 subjects) or the OSA group (2,260 subjects), respectively. Subjects who did not meet the insomnia criteria and had an AHI < 15 e/h were included in the No-OSA group (2,738 subjects), which served as the reference population for this study. As explained, these criteria for establishing Insomnia, OSA, and COMISA groups have been employed in several works analyzing the SHHS database^24,25,29^. Table 1 presents the demographic and relevant polysomnographic data of the study population.

**Table 1:** Demographic and clinical characteristics of the population under study.

	No-OSA	Insomnia	OSA	COMISA	*p*-value
Subjects (*n*)	2738 (51.3%)	190 (3.6%)	2260 (42.4%)	147 (2.3%)	-
Males (*n*)	977 (35.7%)	37 (19.5%)	1431 (63.3%)	81 (55.1%)	-
Age (years)	61.0 [17.0] ^(b)^	59.0 [16.0] ^(d)^	66.0 [16.0] ^(b, d)^	64.0 [15.0]	< 0.01
BMI (kg/m^2^)	26.3 [5.6] ^(b, c)^	27.1 [6.1] ^(d, e)^	28.8 [6.6] ^(b, d)^	29.9 [7.4] ^(c, e)^	< 0.01
AHI (e/h)	7.4 [6.4] ^(b, c)^	7.6 [7.0] ^(d, e)^	25.6 [18.1] ^(b, d)^	25.4 [20.2] ^(c, e)^	< 0.01
Sleep Latency (min)	47.5 [48] ^(a)^	59.0 [56.4] ^(a, d)^	48 [47.5] ^(d)^	51.3 [56]	< 0.01
Awakenings (*n*)	24.0 [13.0] ^(b, c)^	22.0 [13.0] ^(d, e)^	29.5 [19.0] ^(b, d)^	27.0 [19.0] ^(c, e)^	< 0.01
WASO (min)	43.5 [44.0] ^(b, c)^	45.5 [54.5] ^(d, e)^	58.0 [58.0] ^(b, d)^	59.0 [65.0] ^(c, e)^	< 0.01
TAI (e/h)	13.9 [7.9] ^(b, c)^	13.9 [8.4] ^(d, e)^	22.2 [13.3] ^(b, d)^	19.2 [13.0] ^(c, e)^	< 0.01
TST (min)	376.5 [81.5] ^(a, b,c)^	361.5 [106.5] ^(a)^	357.5 [84.5] ^(b)^	340.9 [89.5] ^(c)^	< 0.01
TNREM (min)	295.5 [65.0] ^(b, c)^	294.5 [83.5]	289.0 [68.00] ^(b)^	286.0 [81.9] ^(c)^	< 0.01
TREM (min)	77.0 [39.0] ^(a, b,c)^	70.5 [45.5] ^(a)^	66.5 [37.0] ^(b)^	65.4 [42.0] ^(c)^	< 0.01

Data presented as median [interquartile range] or n (%). *p*-value column refers to the Kruskall-wallis test.

AHI = apnea-hypopnea index; Awakenings = number of awakenings during night; BMI = body mass index; WASO = Time spent in wake after sleep onset; TAI: Total Arousal Index; OSA = Obstructive Sleep Apnea; COMISA = Comorbid insomnia and sleep apnea; TST = total sleep time; TNREM = time spent in non-rapid eye movement sleep stage; TREM = time spent in rapid eye movement sleep stage.

(a): Statistically significant differences (*p* < 0.01 after applying FDR correction) between No-OSA and Insomnia.

(b): Statistically significant differences (*p* < 0.01 after applying FDR correction) between No-OSA and OSA.

(c): Statistically significant differences (*p* < 0.01 after applying FDR correction) between No-OSA and COMISA.

(d): Statistically significant differences (*p* < 0.01 after applying FDR correction) between Insomnia and OSA.

(e): Statistically significant differences (*p* < 0.01 after applying FDR correction) between Insomnia and COMISA.

(f): Statistically significant differences (*p* < 0.01 after applying FDR correction) between OSA and COMISA.

### Signal preprocessing and HRV extraction

In this study, we performed HRV analysis derived from ECG signals. As previously mentioned, ECG was acquired during PSG at frequencies of 125–250 Hz. The initial step in HRV analysis involved applying an R-peak detection algorithm based on the Hilbert transform, as proposed by Benitez et al.^30^, which has been previously utilized in the OSA context^31–33^. To address erroneous heartbeat detection, we applied an artifact rejection procedure to remove physiologically implausible peaks using the following criteria^31–33^: (i) the duration of R-R intervals was constrained to between 0.33s and 1.5s, and (ii) the maximum allowed difference between two consecutive R-R intervals was 0.66s. By applying these criteria, we removed periods of signal disconnections (few heartbeats detected), as well as noisy segments where false peaks could be introduced, for example, by motion artifacts. This led us to obtain the normal-to-normal (N-N) intervals time series, which estimates the time between sinoatrial node heartbeats. Additionally, to ensure enough representative nocturnal data, we excluded subjects with preprocessed HRV recordings shorter than three hours^28,32^.

### Characterization of nocturnal ANS behavior

In the original SHHS study, sleep stages were scored according to the guidelines developed by Rechtschaffen and Kales^28,34^. Given the importance of distinguishing between wake and sleep stages in the context of insomnia, each individual sample throughout the entire recordings of each subject was assigned to either wake or sleep. HRV characterization was then conducted for the whole night (WN), as well as separately for wake and sleep stages. To complement our analysis, HRV characterization across non-rapid eye movement (NREM) and rapid eye movement (REM) sleep stages is also provided in the Supplementary Information.

#### Characterization in the Temporal domain

Conventional HRV metrics in the time domain provide valuable insights into overall variability and short-term HRV fluctuations, and they are commonly used to evaluate ANS function and cardiovascular health^35^. For this purpose, we computed four widely assessed time-domain HRV metrics^35^:

- Mean heart rate (*mHR*), measured in beats per minute. This measure can reflect both changes caused by SNS activity (increasing heart rate), and parasympathetic nervous system (PNS) activity (decreasing heart rate).
- Mean of the standard deviation of N-N intervals (*SDNNI*), which reflects average short-term HRV, is influenced by both SNS and PNS activity.
- Root Mean Square of successive differences of N-N intervals (*RMSSD*), primarily influenced by PNS activity.
- Percentage of adjacent N-N intervals differing over 50ms (*pNN50*), also correlated with PNS activity.

#### Characterization in the frequency domain

The extraction of N-N intervals results in an irregularly sampled signal. Therefore, prior to frequency domain analysis, it is necessary to resample the HRV signals. Accordingly, consistent with previous works, each HRV signal was resampled to a constant frequency rate of 3.41 Hz^31,32^. Once all recordings were uniformly sampled, the continuous wavelet transform (CWT) was computed using a complex Morlet wavelet at scales corresponding to the range 0.0005–0.4 Hz^36^. Unlike conventional spectral analysis, CWT allowed us to: (i) evaluate very low-frequency regions not reached by other common spectral HRV analyses, which is advantageous in cases of insomnia where the macro-sleep structure is altered^2^; and (ii) obtain the estimation of power spectral density (PSD) at each time instant, linked to the location of each N-N interval sample. Therefore, we can associate a specific sleep stage with each instant where the PSDs are estimated, which is of paramount importance in sleep recordings with many transitions between wake and the different sleep stages, as is the case with OSA^2^. These PSDs were subsequently normalized (PSDns) at each temporal instant. Thus, to characterize HRV activity in the frequency domain, we extracted spectral features from the averaged PSDns across the entire recording (WN), as well as separately for wake (combining pre-sleep and intermediate wake periods) and sleep stages.

Traditional HRV frequency analysis focuses on three frequency ranges: very low frequency (VLF: 0–0.04 Hz), low frequency (LF: 0.04–0.15 Hz), and high frequency (HF: 0.14–0.4 Hz)^35^. However, in the context of pediatric OSA, we previously demonstrated that these fixed boundaries, defined based on normal ANS behavior, have limitations in evaluating specific ANS alterations^32,33^. To address this issue, we developed a procedure to identify specific frequency ranges relevant to pediatric OSA, allowing for a more accurate assessment of disease-related alterations^32^. While the existence of a specific frequency range has also been suggested in OSA adults^31,37^, a concrete methodology has not yet been applied in this population. Therefore, we have replicated here our previously reported methodology in Martin-Montero et al.^32^ to identify specific frequency ranges in the context of COMISA in adults.

The methodology for extracting specific frequency ranges involved two approaches: one within 0–0.15 Hz range and another within 0.15–0.4 Hz range (HF, also known as the respiratory frequency range). This second approach accounts for age-related differences in respiration within the HF range, enabling an individualized adaptive analysis^32^. Accordingly, we directly adopted the specific frequency range identified between 0.15 and 0.4 Hz, named BWRes, which is calculated as a 0.04 Hz range around the individual respiratory peak within the HF range^32^. Regarding the 0–0.15 Hz range, we replicated the original methodology^32^ to identify COMISA-specific HRV frequency ranges. This involved applying the Mann-Whitney *U*-test to compare, frequency by frequency, amplitude values from the average PSDns across WN between each pair of groups. Specific frequency ranges were defined as those where at least two comparisons exceeded the significance threshold of *p*-value < 0.01 after applying false discovery rate (FDR) correction. We selected FDR over other more conservative alternatives, such as the Bonferroni correction, due to the high number of comparisons included. For a detailed description of this methodology, refer to Martin-Montero et al.^32^.

Following the identification of the bands of interest for our research, we conducted the frequency domain analysis by computing relative power (*RP*, the sum of PSDn amplitude values within specified frequency ranges) across both classic and novel frequency bands.

### Statistical analysis

Since the computed features did not fit homoscedasticity nor normality test, we applied the non-parametric Mann-Whitney *U*-test to evaluate differences between groups and to define specific frequency ranges, as mentioned earlier. The significance threshold for the study was set at *p*-values < 0.01 after applying FDR correction. As can be observed in the population distribution, there is a clear imbalance between the populations included in the COMISA and Insomnia groups compared to the No-OSA and OSA groups. To assess the influence of this imbalance on the statistical significance of the differences, a permutation test procedure was applied. A detailed description of this methodology is provided in the Supplementary Information, and the statistical results are shown in Supplementary Table S1.

Additionally, a descriptive analysis of the feature distributions for each group was represented using notched boxplots for all features where at least one statistically significant difference between two groups was observed.

## Results

### Identification of spectral band of interest

**Fig. 1:**
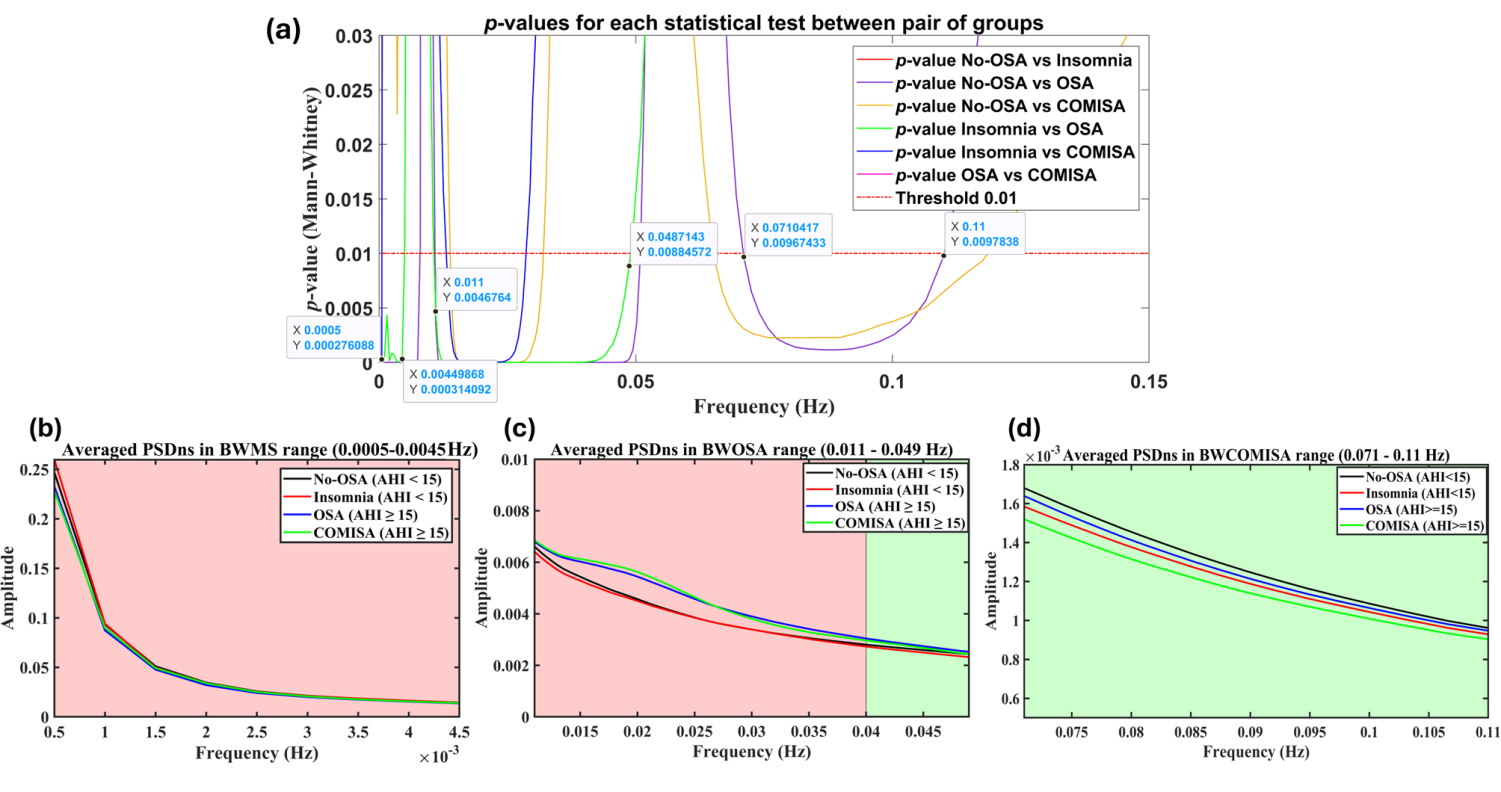
Search of specific frequency bands in the range 0–0.15 Hz: (a) *p*-values for each comparison between groups from the Mann-Whitney *U*-test after applying FDR correction, with the black markers highlighting regions where at least two comparisons were statistically significant; (b) Averaged PSDns in the BWMS band (0.0005–0.0045 Hz), with higher activity; (c) Averaged PSDns in the BWOSA band (0.011–0.049 Hz); (d) Averaged PSDns in the BWCOMISA band (0.071–0.11 Hz).

The seek for specific frequency ranges resulted in the identification of three frequency bands that exhibited statistical differences between at least two groups. Figure 1 illustrates the results of this statistical analysis (Fig. 1a) and the behavior of the averaged PSDns for each group within the selected ranges (Figs. 1b-d). The first frequency band, spanning 0.0005–0.0045 Hz, corresponds to a range identified in our previous work that reflects macro-sleep structure^32^. Accordingly, we have renamed this band as BWMS. It can be appreciated that the Insomnia and No-OSA groups show higher activity within BWMS, while it is reduced in OSA and COMISA patients, especially near 0.0005 Hz (Fig. 1b). The second frequency band, covering 0.011–0.049 Hz, captures recurrence patterns between 20 and 90s, consistent with the periodicity of apneic events in adults^38^. This spectral band corresponds to the OSA frequency range identified in our previous work^32^, but shifted to the specific apneic recurrence patterns in adults. As expected, activity in this range is higher for OSA and COMISA patients (Fig. 1c), so we have named it BWOSA. The third frequency band, a novel finding of this study, spans 0.071–0.11 Hz. Characterized by reduced activity in the COMISA group (Fig. 1d), we have designated this band as BWCOMISA.

Consequently, the spectral features computed during WN, wake, and sleep stages included *RP*_*VLF*_, *RP*_*LF*_, *RP*_*HF*_, *RP*_*BWMS*_, *RP*_*BWOSA*_, *RP*_*BWCOMISA*_, *RP*_*BWRes*_, as well as the normalized power in LF (*LFn*).

### Differences in the CWTs between types of subjects

**Fig. 2:**
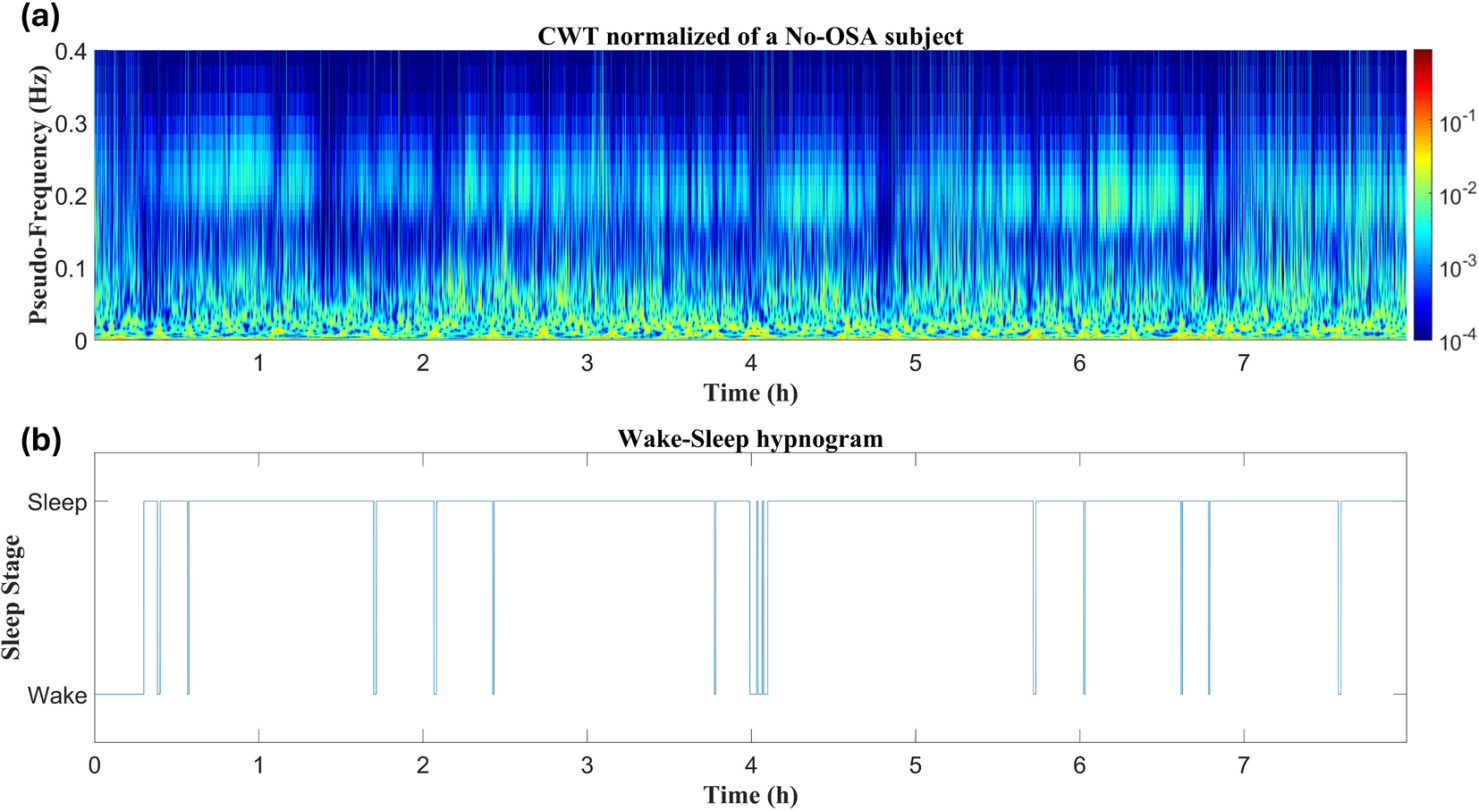
The HRV time-frequency distribution across the night of a No-OSA subject: (a) Normalized CWT computed for the entire register; (b) Wake-sleep hypnogram. The subject presents higher power concentration at low frequencies (below 0.1 Hz), and high activity within HF range, corresponding to normal respiratory activity during sleep.

**Fig. 3:**
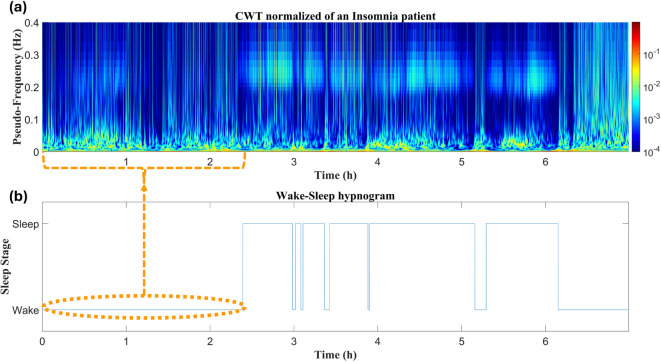
The HRV time-frequency distribution across night of an Insomnia patient: (a) Normalized CWT computed for the entire register; (b) Wake-sleep hypnogram. Highlighted orange area reflects sleep latency on the Insomnia patient, during which power is concentrated at BWMS frequency range. Once sleep begins, the power distribution resembles more to the No-OSA subject, and there is also high respiratory activity within HF.

**Fig. 4:**
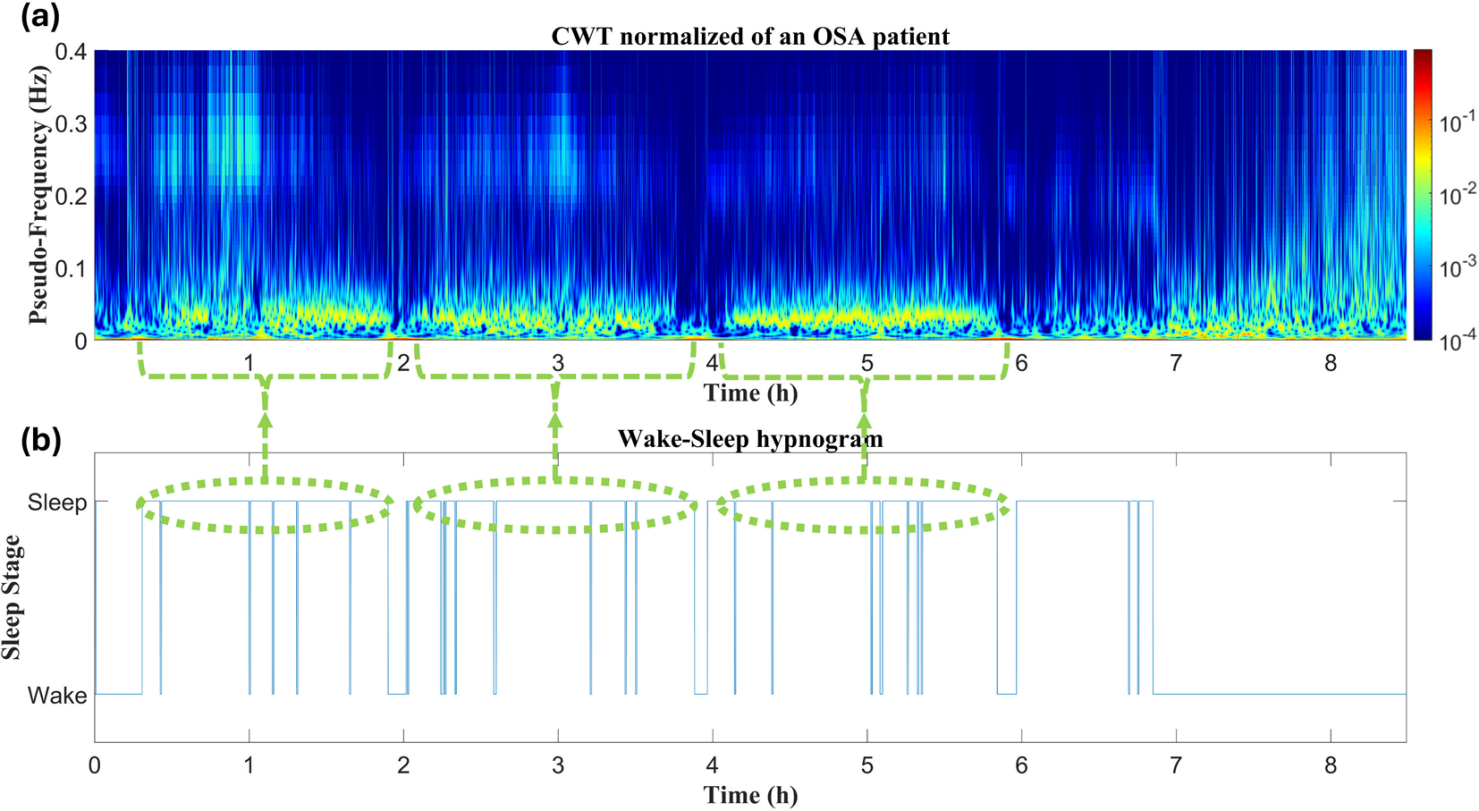
The HRV time-frequency distribution across night of an OSA patient: (a) Normalized CWT computed for the entire register; (b) Wake-sleep hypnogram. Highlighted green areas reflect sleep periods during which apneic events take place. During sleep, power concentration occurs mainly at BWOSA frequencies, rather than in the HF range as in the Insomnia and No-OSA case.

**Fig. 5:**
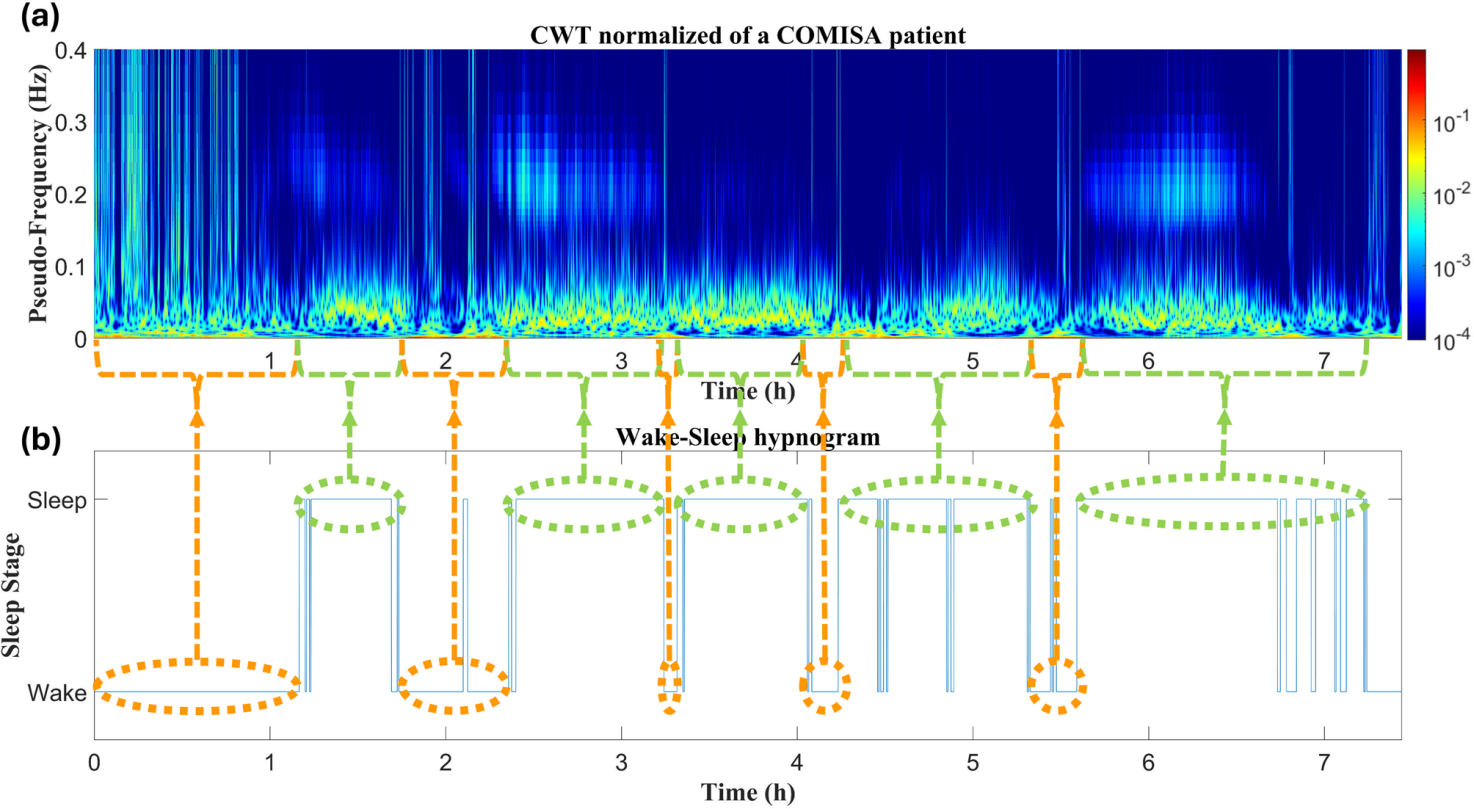
The HRV time-frequency distribution across night of a COMISA patient: (a) Normalized CWT computed for the entire register; (b) Wake-sleep hypnogram. Highlighted orange areas reflect wake periods, during which power spreads to the whole frequency range. Highlighted green areas reflect sleep periods, during which power concentration shifts to BWOSA frequencies. The patient presents several sleep disruptions, taking longer time to return to sleep compared to the other groups.

Illustrative examples of normalized CWTs computed for a patient from each group (No-OSA, Insomnia, OSA, and COMISA) are depicted in Figs. 2, 3, 4 and 5. Figure 2 illustrates the time-frequency evolution for a No-OSA subject (Fig. 2a), alongside the wake-sleep hypnogram throughout the night (Fig. 2b). It can be observed that there is a higher power concentration at low frequencies (below 0.1 Hz), with additional activity within the HF range (0.15–0.4 Hz), corresponding to normal respiratory activity during sleep. Notably, there are few transitions from sleep to wake after sleep onset.

Figure 3 depicts the case of an Insomnia patient. A notable feature is the elevated sleep onset latency, exceeding two hours, during which power is mainly concentrated at very low frequencies (near 0.0005 Hz, Fig. 3a), corresponding to the BWMS frequency band. Once sleep begins, the power distribution becomes more similar to that of the No-OSA subject, with fewer sleep disruptions, though the disruptions that do occur are longer than those in the No-OSA subject. Additionally, there is evident respiratory activity within the HF range.

Figure 4 shows the overnight time-frequency evolution for an OSA patient. During sleep periods, power is concentrated below 0.05 Hz, corresponding to BWOSA frequencies, and the amount of power across the HF range is markedly lower compared to Insomnia and No-OSA subjects. Additionally, there are more frequent wake-sleep transitions after sleep onset.

Finally, Fig. 5 presents the HRV time-frequency distribution for a COMISA subject (Fig. 5a), along with the corresponding hypnogram (Fig. 5b). It took over an hour for the subject to fall asleep, with power spreading across the entire frequency range. Once sleep begins, power concentration is primarily focused on BWOSA frequencies. Additionally, there are several sleep disruptions, with the patient taking longer to return to sleep compared to the other cases. During these disruptions, power concentration shifts to lower frequencies than those observed during sleep.

### Descriptive analysis of the features

**Fig. 6:**
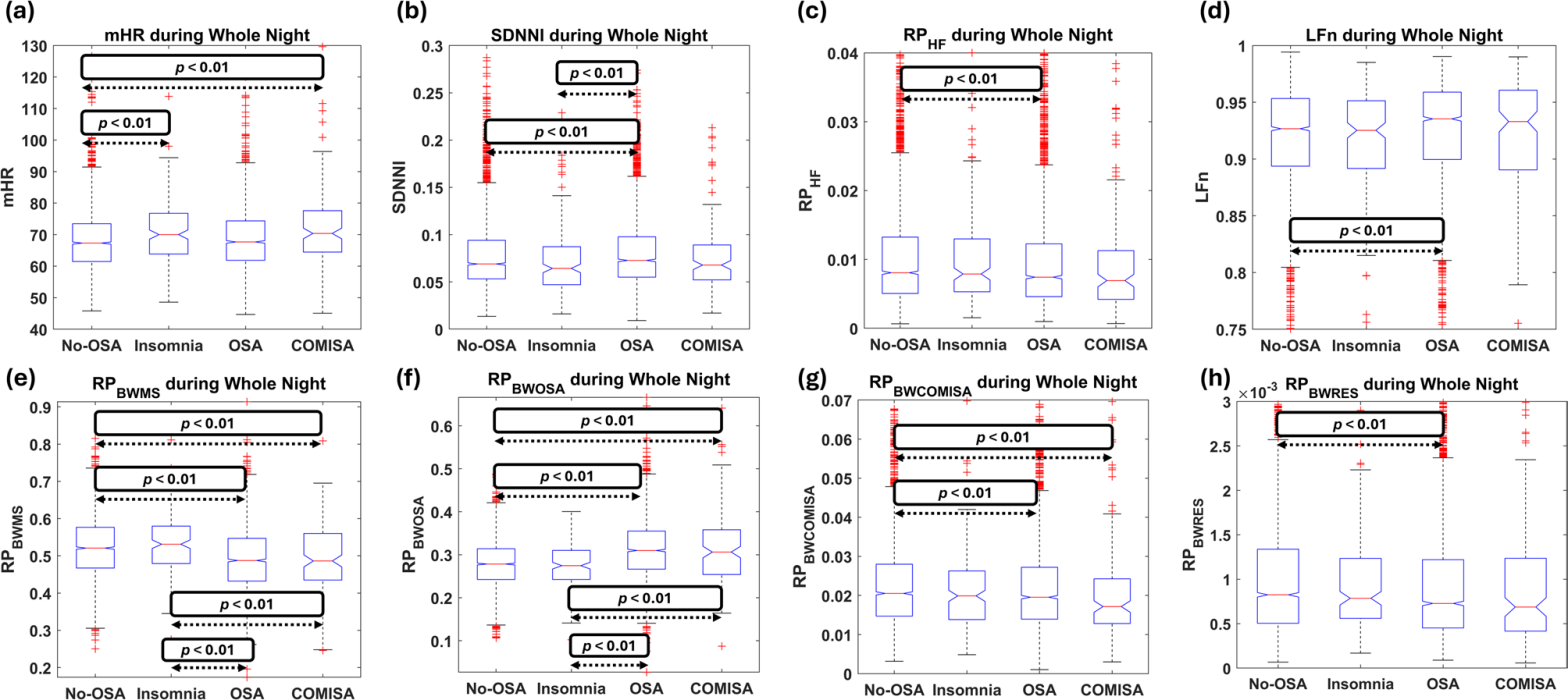
Boxplot distribution of the features computed across the whole night that reached statistically significant differences between any group comparisons. Statistically significant differences are highlighted within each corresponding subplot, with arrows delimiting where the differences arise. (a) *mHR* boxplots; (b) *SDNNI* boxplots; (c) *RP*_*HF*_ boxplots; (d) *LFn* boxplots; (e) *RP*_*BWMS*_ boxplots; (f) *RP*_*BWOSA*_ boxplots; (g) *RP*_*BWCOMISA*_ boxplots; (h) *RP*_*BWRes*_ boxplots.

**Fig. 7:**
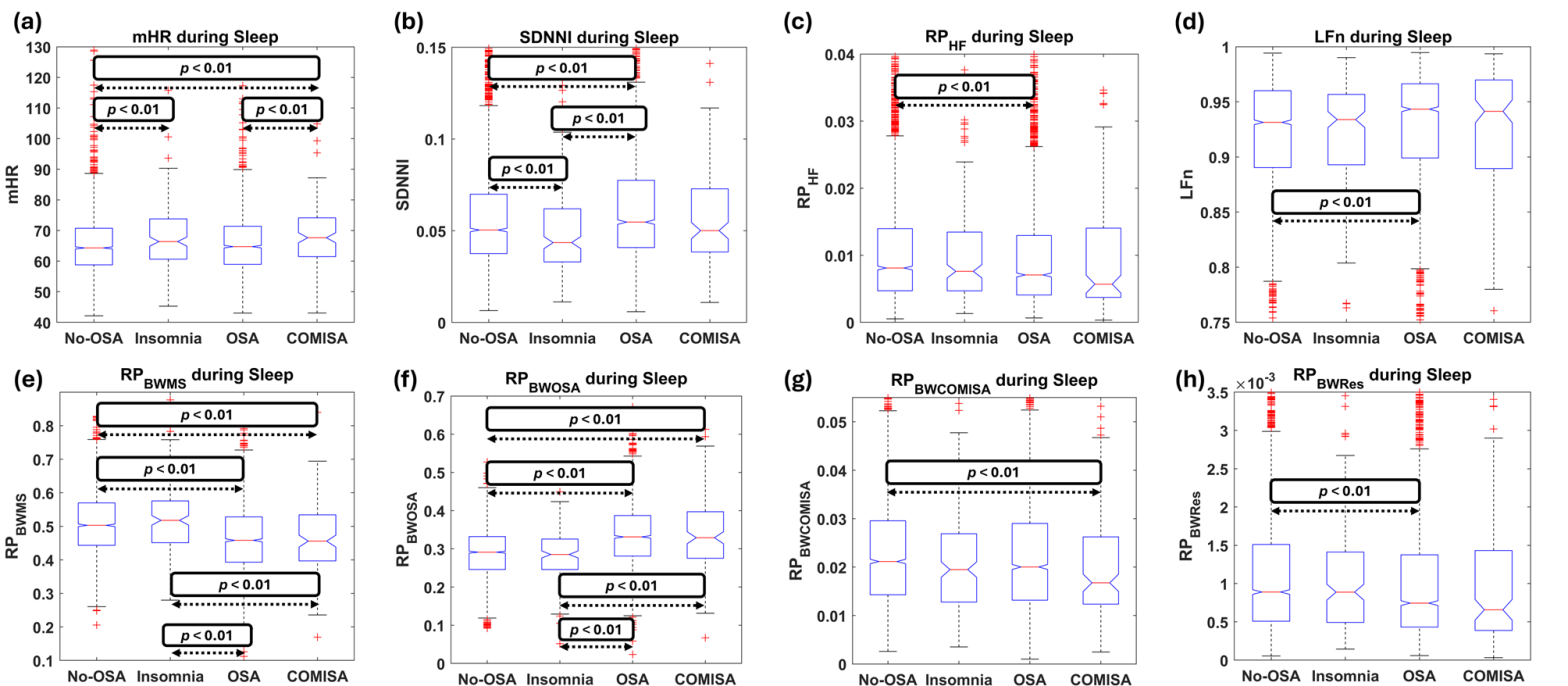
Boxplot distribution of the features computed across sleep periods that reached statistically significant differences between any group comparisons. Statistically significant differences are highlighted within each corresponding subplot, with arrows delimiting where the differences arise. (a) *mHR* boxplots; (b) *SDNNI* boxplots; (c) *RP*_*HF*_ boxplots; (d) *LFn* boxplots; (e) *RP*_*BWMS*_ boxplots; (f) *RP*_*BWOSA*_ boxplots; (g) *RP*_*BWCOMISA*_ boxplots; (h) *RP*_*BWRes*_ boxplots.

**Fig. 8:**
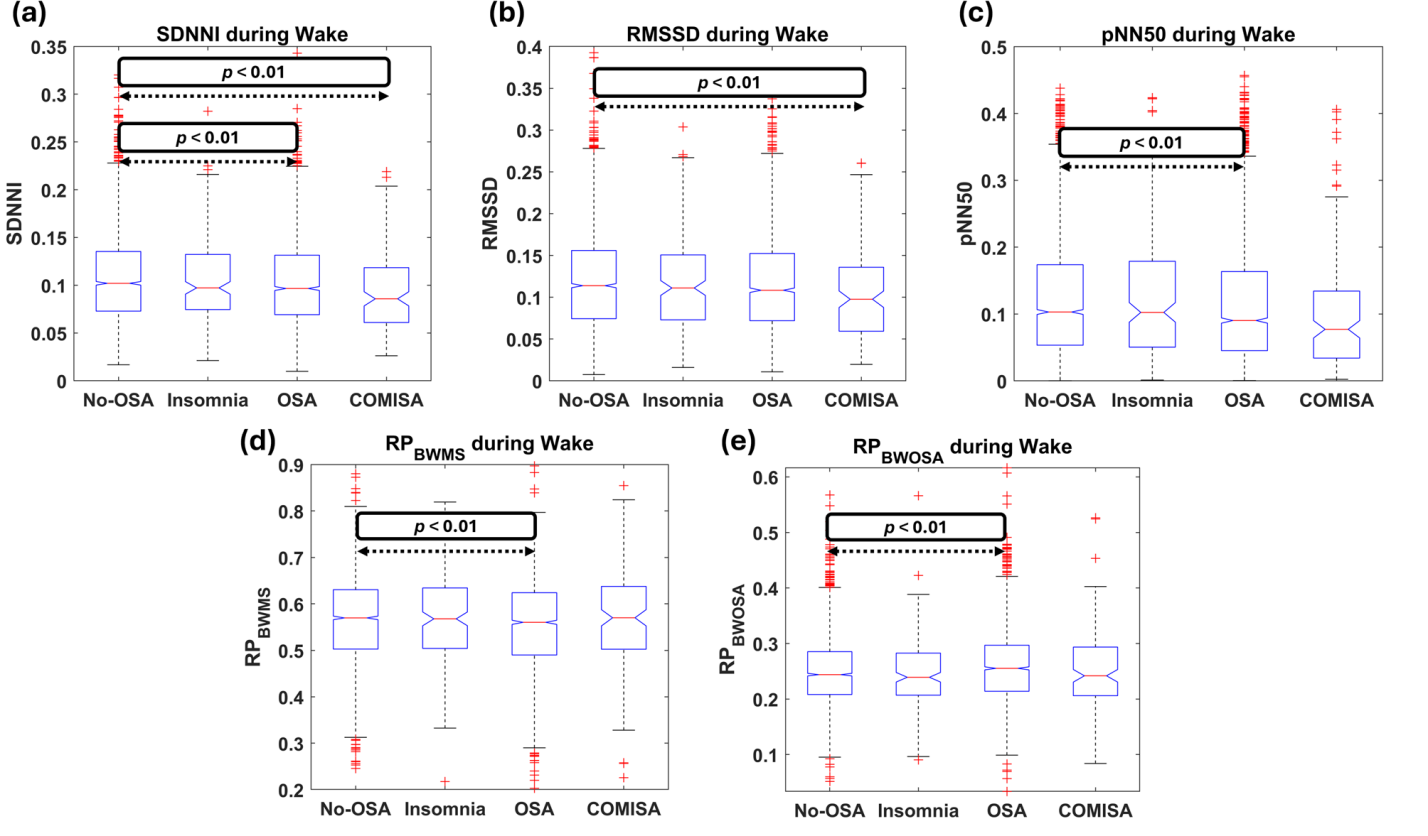
Boxplot distribution of the features computed across wake periods that reached statistically significant differences between any group comparisons. Statistically significant differences are highlighted within each corresponding subplot, with arrows delimiting where the differences arise. (a) *SDNNI* boxplots; (b) *RMSSD* boxplots; (c) *pNN50* boxplots; (d) *RP*_*BWMS*_ boxplots; (e) *RP*_*BWOSA*_ boxplots.

Table 2 presents the *p*-values obtained from the comparisons between HRV features across the WN, representing overall HRV nocturnal behavior, as well as during wake and sleep periods. To complement these results and assess how the differences emerge, the boxplot distributions of the features showing any statistically significant differences across WN, wake, and sleep periods are depicted in Figs. 6, 7 and 8, respectively.

**Table 2:** Statistically significant differences derived from the Mann-Whitney *U*-test in HRV features between each pair of groups considered, following FDR correction and permutation tests.

Differences in HRV features computed across whole night						
Feature	No-OSA vs. Insomnia	No-OSA vs. OSA	No-OSA vs. COMISA	Insomnia vs. OSA	Insomnia vs. COMISA	OSA vs. COMISA
*mHR*	**< 0.01**	n.s.	**< 0.01**	n.s.*	n.s.	n.s.*
*SDNNI*	n.s.	**< 0.01**	n.s.	**< 0.01**	n.s.	n.s.
*RMSSD*	n.s.	n.s.	n.s.	n.s.	n.s.	n.s.
*pNN50*	n.s.	n.s.	n.s.	n.s.	n.s.	n.s.
*RP*_*VLF*_	n.s.	n.s.	n.s.	n.s.	n.s.	n.s.
*RP*_*LF*_	n.s.	n.s.	n.s.	n.s.	n.s.	n.s.
*RP*_*HF*_	n.s.	**< 0.01**	n.s.	n.s.	n.s.	n.s.
*LFn*	n.s.	**< 0.01**	n.s.	n.s.	n.s.	n.s.
*RP*_*BWMS*_	n.s.	**< 0.01**	**< 0.01**	**< 0.01**	**< 0.01**	n.s.
*RP*_*BWOSA*_	n.s.	**< 0.01**	**< 0.01**	**< 0.01**	**< 0.01**	n.s.
*RP*_*BWCOMISA*_	n.s.	**< 0.01**	**< 0.01**	n.s.	n.s.	n.s.
*RP*_*BWRes*_	n.s.	**< 0.01**	n.s.	n.s.	n.s.	n.s.

Differences in HRV features computed across wake periods						
Feature	No-OSA vs. Insomnia	No-OSA vs. OSA	No-OSA vs. COMISA	Insomnia vs. OSA	Insomnia vs. COMISA	OSA vs. COMISA
*mHR*	n.s.	n.s.	n.s.	n.s.*	n.s.	n.s.
*SDNNI*	n.s.	**< 0.01**	**< 0.01**	n.s.	n.s.	n.s.*
*RMSSD*	n.s.	n.s.	**< 0.01**	n.s.	n.s.	n.s.
*pNN50*	n.s.	**< 0.01**	n.s.*	n.s.	n.s.	n.s.
*RP*_*VLF*_	n.s.	n.s.	n.s.	n.s.	n.s.	n.s.
*RP*_*LF*_	n.s.	n.s.	n.s.	n.s.	n.s.	n.s.
*RP*_*HF*_	n.s.	n.s.	n.s.	n.s.	n.s.	n.s.
*LFn*	n.s.	n.s.	n.s.	n.s.	n.s.	n.s.
*RP*_*BWMS*_	n.s.	**< 0.01**	n.s.	n.s.	n.s.	n.s.
*RP*_*BWOSA*_	n.s.	**< 0.01**	n.s.	n.s.	n.s.	n.s.
*RP*_*BWCOMISA*_	n.s.	n.s.	n.s.	n.s.	n.s.	n.s.
*RP*_*BWRes*_	n.s.	n.s.	n.s.	n.s.	n.s.	n.s.

Differences in HRV features computed across sleep periods						
Feature	No-OSA vs. Insomnia	No-OSA vs. OSA	No-OSA vs. COMISA	Insomnia vs. OSA	Insomnia vs. COMISA	OSA vs. COMISA
*mHR*	**< 0.01**	n.s.	**< 0.01**	n.s.*	n.s.	**< 0.01**
*SDNNI*	**< 0.01**	**< 0.01**	n.s.	**< 0.01**	n.s.	n.s.
*RMSSD*	n.s.	n.s.	n.s.	n.s.	n.s.	n.s.
*pNN50*	n.s.	n.s.	n.s.	n.s.*	n.s.	n.s.
*RP*_***VLF***_	n.s.	n.s.	n.s.	n.s.	n.s.	n.s.
*RP*_***LF***_	n.s.	n.s.	n.s.	n.s.*	n.s.	n.s.
*RP*_***HF***_	n.s.	**< 0.01**	n.s.	n.s.	n.s.	n.s.
*LFn*	n.s.	**< 0.01**	n.s.	n.s.	n.s.	n.s.
*RP*_***BWMS***_	n.s.	**< 0.01**	**< 0.01**	**< 0.01**	**< 0.01**	n.s.
*RP*_***BWOSA***_	n.s.	**< 0.01**	**< 0.01**	**< 0.01**	**< 0.01**	n.s.
*RP*_***BWCOMISA***_	n.s.	n.s.	**< 0.01**	n.s.	n.s.	n.s.
*RP*_***BWRes***_	n.s.	**< 0.01**	n.s.	n.s.	n.s.	n.s.

n.s.: non-significant (p-value > 0.01).

*Deemed as non-significant after permutation tests. See Supplemental Information.

Statistically significant comparisons (p-value < 0.01 after FDR correction) appear in bold.

Regarding temporal features across WN, *mHR* was elevated, apparently due to insomnia effects (Fig. 6a), with statistically significant differences observed between both Insomnia and COMISA patients compared to the No-OSA group. Additionally, *SDNNI* was higher in the OSA group compared to both the Insomnia and No-OSA groups, but not significantly different from the COMISA population (Table 2; Fig. 6b). When evaluating frequency domain features, the specific HRV features developed in this study exhibited greater differences between groups than the classic HRV features. While *RP*_*BWMS*_ and *RP*_*BW*__*OSA*_ showed the most pronounced differences, *RP*_*VLF*_ and *RP*_*LF*_ did not reach statistical significance between any groups (neither in WN, nor during sleep or wake periods). The differences in *RP*_*BWMS*_ and *RP*_*BW*__*OSA*_ seem to arise as an OSA consequence, with notably reduced activity in BWMS and increased activity within BWOSA for OSA and COMISA groups compared to No-OSA and Insomnia patients (Fig. 6e and f). The No-OSA group displayed higher activity within HF and BWRes (Fig. 6h) bands, as well as reduced *LFn* compared to the OSA group, and lower activity across BWCOMISA (Fig. 6g) compared to both OSA and COMISA patients.

When examining features during wake, it can be observed that most of the significant differences observed across WN in the frequency domain disappeared, with temporal domain features becoming more relevant during this period. Specifically, reduced *SDNNI* was observed in the OSA and COMISA groups compared to the No-OSA group, as well as reduced *pNN50* in OSA vs. No-OSA (Fig. 7a and c, respectively), while RMSSD was only altered in COMISA patients, showing lower values than the No-OSA group (Fig. 7b). In the frequency domain, the only features that reached statistically significant differences were reduced *RP*_*BWMS*_ and increased *RP*_*BWOSA*_ in the OSA group (but not in the COMISA group) compared to the No-OSA subjects (Fig. 7d and e).

Finally, regarding HRV features during sleep, the significance of elevated overnight *mHR* in Insomnia was confirmed, with increases observed in both the Insomnia and COMISA groups compared to the No-OSA population. This metric was also increased in the COMISA subjects compared to the OSA group, being the only feature in the study reaching statistically significant difference between these two groups (Table 2; Fig. 8a). *SDNNI* was also altered, showing lower values in Insomnia and higher values in OSA patients compared to the No-OSA group (Fig. 8b). On the other hand, features in the frequency domain regained relevance during these periods. Similar to WN, the most pronounced differences during sleep were observed for the specific features introduced in this study (*RP*_*BWMS*_ and *RP*_*BWOSA*_). The same patterns as in WN were seen in *RP*_*HF*_, *LFn*, *RP*_*BWMS*_, *RP*_*BWOSA*,_ and *RP*_*BWRes*_ (Fig. 8e, f and h). The only differentiation in frequency domain features during sleep compared to WN was for *RP*_*BWCOMISA*_, where the differences between OSA and No-OSA subjects disappeared, leaving the COMISA group as the only population showing altered BWCOMISA activity (Fig. 8g).

## Discussion

In this study, a time and frequency analysis of overnight HRV signals, differentiating between wake and sleep periods, was performed for the first time in the context of COMISA. By evaluating both classic and novel HRV metrics, specific alterations resulting from the coexistence of insomnia and OSA were identified. The primary alterations in the COMISA population were found to be consequences of OSA during sleep; however, insomnia also significantly affected HRV activity at night, primarily in the temporal domain. These findings underscore the importance of jointly considering the influences of insomnia and OSA on ANS dysfunction and their cardiovascular implications during the night through HRV analysis.

### ANS alterations due to COMISA

The approach followed revealed specific alterations in COMISA patients across the three evaluated periods. To support and contextualize the discussion of the major findings, Table 3 presents the main results that highlight the specific HRV patterns observed in the patients under study. Regarding WN features, the most significant alterations in COMISA patients were found in BWMS and BWOSA activity, thus allowing clear differentiation of their behavior from the HRV of Insomnia or No-OSA patients (see Tables 2 and 3, and Fig. 6). In our previous work, where we first identified ranges similar to BWMS and BWOSA in the context of pediatric OSA, we observed that BWMS activity decreased as OSA severity increased, with activity shifting to BWOSA frequencies, which capture sympathetic excitation due to recurrent apneic events^32^. Figures 2, 3, 4 and 5 illustrate that during wake periods, power is mainly concentrated around BWMS frequencies in all patient types. However, during sleep, there is a clear differentiation in power concentration between the No-OSA and Insomnia groups compared to OSA and COMISA patients. While individuals with an AHI < 15 e/h maintain power concentration at BWMS frequencies, patients meeting OSA criteria exhibit a shift in power distribution to BWOSA activity, resulting in the marked differences observed in BWMS and BWOSA (Table 2). Additionally, previous studies suggested that, while primary Insomnia patients have difficulty initiating sleep, the COMISA population is more likely to experience difficulties maintaining sleep^9,39^. This pattern was also observed in our study (Table 1) as Insomnia patients have higher sleep latency, but COMISA and OSA patients experience greater awakenings and WASO. These differences in macro-sleep structure may also underlie the reduced BWMS activity in OSA and COMISA patients. Collectively, these results suggest that the primary overnight ANS dysfunction in COMISA patients arises as an OSA consequence, manifested as SNS activation during apneic events and sleep disruptions and leading to increased BWOSA and reduced BWMS activity. Furthermore, while reduced BWCOMISA and BWRes activity appear to be characteristic of OSA patients in comparison with the No-OSA group, only BWCOMISA reductions emerge in the COMISA population. Another feature that appears to play an important role in the COMISA population across WN is *mHR*. Similar to the main conclusions drawn from frequency features, an increased *mHR* reflects sympathetic excitation^35^. However, in this case, the differences in *mHR*, observed when comparing the No-OSA group with the Insomnia and COMISA groups, suggest that the SNS alterations captured by the temporal analysis arise as a consequence of insomnia in the context of COMISA.

**Table 3:** Summary of the major findings regarding specific HRV alterations identified in each patient group. BWOSA and BWMS exhibited the most pronounced differences throughout the study. In addition, specific HRV alterations associated with the COMISA and Insomnia populations are also highlighted.

Patients group	Major findings
COMISA	Increased ***RP***_***BWOSA***_ and reduced ***RP***_***BWMS***_ during whole night
	Increased ***mHR*** and reduced ***RP***_***BWCOMISA***_ at sleep, and low ***RMSSD*** during wake
OSA	Increased ***RP***_***BWOSA***_ and reduced ***RP***_***BWMS***_ during whole night
	Normal ***mHR*** and ***RP***_***BWCOMISA***_ activity during sleep
INSOMNIA	Normal ***RP***_***BWOSA***_ and ***RP***_***BWMS***_ activity during whole night
	Increased ***mHR*** and reduced ***SDNNI*** during sleep

*RP*_*BWOSA*_: Relative Power computed within BWOSA frequency range (0.011–0.049 Hz); *RP*_*BWMS*_: Relative Power computed within BWMS frequency range (0.0005–0.0045 Hz); *mHR*: mean Heart Rate; *RMSSD*: Root mean square of the successive differences of N-N intervals; *SDNNI*: mean of the standard deviation of N-N intervals.

Regarding wake periods, the significance of frequency analysis in the COMISA population seems to diminish, as none of the frequency features reached statistically significant differences in this group. Interestingly, it is not the presence of differences but their absence that draws attention. While *RP*_*BWMS*_ and *RP*_*BWOSA*_, which showed the most differentiation for COMISA patients across WN, did not reach statistically significant differences during wake periods, these features remain altered in the OSA population compared to the No-OSA group. Previous studies have demonstrated that sympathetic activation associated with apneic events persists during waking periods in OSA patients^14,40^. This may imply that while the OSA group maintains altered HRV activity in these frequency ranges during wake periods, this is not the case for the COMISA population. Conversely, temporal features gain greater significance during wake periods, particularly when comparing HRV activity of the No-OSA group with that of the OSA and COMISA populations. Autonomic dysfunction during wake, evidenced by reduced *SDNNI*, appears in both OSA and COMISA groups, while PNS depression, indicated by reduced *RMSSD*, is only observed in COMISA patients. This finding highlights another specific ANS alteration in the context of COMISA.

Finally, the analysis across sleep periods confirmed the crucial role of *mHR* in evaluating the effects of insomnia and revealed that frequency analysis is essential for assessing the impact of OSA during these periods. Notably, while *mHR* did not show statistically significant differences during wakefulness, it emerged as the most relevant temporal feature during sleep, being the only feature across the entire study where differences between OSA and COMISA were observed. This can be attributed to the inherent sympathetic excitation during wakefulness, which increases heart rate in all groups compared to sleep periods^41^. However, Insomnia and COMISA patients experienced more wake periods throughout the night, hampering heart rate reduction during sleep, unlike the other groups. This elevated *mHR* is more pronounced in COMISA patients, as differences with the OSA group did not appear in the Insomnia population. The increased *mHR* during sleep is consistent with the findings of Spiegelharder et al.^42^, who reported a reduced wake-to-sleep HR decrease in insomnia patients. This phenomenon is attributed to a hyper-arousal state before sleep and the anxiety surrounding poor sleep, which is exacerbated by longer sleep latencies, acting as a significant stressor in individuals with insomnia^42,43^. These observations highlight insomnia as a key contributor to the increased *mHR* in the COMISA population.

Relative to frequency analysis, the conclusions drawn from *RP*_*BWMS*_ and *RP*_*BWOSA*_ across the WN can be extended to sleep periods, underscoring the altered SNS activity due to recurring apneic events as the main ANS dysfunction in the COMISA population. However, a distinction emerges between WN and sleep when evaluating *RP*_*BWCOMISA*_ in OSA and COMISA patients. While both OSA and COMISA showed decreased BWCOMISA activity compared to the No-OSA group during WN, the differences with the OSA group did not reach statistical significance during sleep periods. As detailed in the Supplemental Information, specific impaired PNS modulation during NREM sleep periods in COMISA patients emerges as the main consequence of this effect. BWCOMISA reflects periodicities between 0.071 and 0.11 Hz. This comprises frequencies within LF (0.04–0.15 Hz), a spectral range associated with SNS and PNS activation, whereby parasympathetic activation has a rapid response time, and sympathetic activation occurs more slowly^44^. Accordingly, it can be stated that BWCOMISA (0.071 to 0.11 Hz) reflects part of the periodic PNS modulation of HRV. It is known that during NREM, there is an intrinsic basal PNS activation^45,46^. This could explain why No-OSA and OSA groups do not show statistically significant differences in BWCOMISA activity during NREM (see Supplemental Table S2), as OSA patients seem to retain the basal PNS function across this frequency range. In contrast, COMISA patients exhibit persistently reduced PNS activity even during NREM (see Supplemental Table S2 and Supplemental Figure S1), suggesting a deeper autonomic dysfunction. On the other hand, during REM, differences emerged in both OSA and COMISA compared to the No-OSA group (see Supplemental Table S2 and Supplemental Figure S2). However, as can be seen in Table 1, the time spent in NREM is considerably longer than in REM. As a result, when analyzing the complete sleep period, the cumulative effect of PNS impairment captured by BWCOMISA becomes statistically significant only in the COMISA group, distinguishing it as a specific HRV characteristic of this population during sleep.

To summarize, we have demonstrated that the combined effects of insomnia and OSA induce specific ANS dysfunction in the COMISA population, creating a distinct fingerprint that can be evaluated through time and frequency HRV analysis. This fingerprint is characterized by reduced *RMSSD* during wake periods, heightened *mHR* during sleep as a consequence of insomnia, and reduced *RP*_*BWMS*_ and *RP*_*BWCOMISA*_ together with increased *RP*_*BWOSA*_ activity during sleep due to OSA effects. These results underscore that the combined impact of both Insomnia and OSA in COMISA patients leads to greater ANS dysfunction than the presence of either sleep disturbance alone.

### Comparison with previous studies

To the best of our knowledge, this is the first work where nocturnal HRV analysis is performed in the context of COMISA. However, some comparisons can be drawn with the unique previous study that analyzed HRV in the COMISA context during the pre-sleep period, conducted by Ma et al.^26^. In that study, which also utilized the SHHS dataset, pre-sleep was defined as the interval between lights-off and sleep onset. When comparing their findings with our results during wake, which includes pre-sleep periods, both studies align in showing the highest *SDNNI* and lowest *pNN50* in the No-OSA group, with these trends reversing in COMISA patients (Fig. 6). Similarly, they also observed no significant differences in HRV measures between Insomnia and COMISA patients (Table 2). Methodological differences between the studies, however, limit further comparisons. Since Ma et al.^26^ did not analyze HRV during sleep, our findings on HRV features in COMISA patients throughout the night cannot be compared with their work or any other existing research. Nevertheless, as stated in the introduction section, ANS dysfunction caused by isolated insomnia and OSA has been extensively studied through HRV analyses, so our results can be discussed in this regard.

Concerning OSA, it is well-established that the cyclical variation of HR due to apneic events leads to ANS dysfunction, characterized by sympathetic predominance and reduced parasympathetic activity during sleep^19,41,47,48^. Our study confirms this through the observed reduction in *RP*_*HF*_ and increase in *LFn* in the OSA population, alongside the rise in *RP*_*BWOSA*_, which is devoted to capturing the recurrent SNS excitation resulting from apneic events^32^. Additionally, ANS dysfunction during wakefulness is reported in our findings through the reduced *SDNNI* in the OSA and COMISA groups, as well as the altered *RP*_*BWMS*_ and *RP*_*BWOSA*_ in OSA patients compared to the No-OSA population, corroborating previous research^40,41,47^.

Regarding insomnia, despite more controversy about ANS impairment^21^, certain outcomes consistently appear in the literature. The most persistent finding is the increased *mHR* compared to healthy population^21,22,43,49–51^. This increase in *mHR* due to insomnia effects has been confirmed in our study (see Table 2; Fig. 6a), with *mHR* being the feature showing the most significant differences between Insomnia and No-OSA groups. Additionally, as previously mentioned, the absence of differences in *mHR* during wakefulness, combined with increased *mHR* during sleep, aligns with the study by Spiegelhalder et al.^42^. Bonnet et al.^49^ conducted one of the pioneering studies on HRV alterations due to insomnia, establishing increased SNS activity and a reciprocal reduction of PNS activity as classic alterations in Insomnia patients. Although some studies have corroborated these results to some extent^22,42,43^, comprehensive replication has been inconsistent^21,22^. In our research, besides *mHR*, the only feature showing ANS alterations in Insomnia patients compared to the No-OSA group was a reduction in *SDNNI* during sleep periods. This reduction due to insomnia has also been reported in the literature^43,49^.

### Limitations and future work

Certain limitations in this research deserve mention. First, although the overall sample size is substantial, there is a noticeable imbalance among the groups. Most participants belong to the No-OSA (*n* = 2,738) and OSA (*n* = 2,260) groups, while the target group, COMISA, comprises the fewest patients (*n* = 147). Despite the potential effects of group imbalance having been tested through the permutation test procedure followed, future research should aim to include a larger number of subjects, particularly in the Insomnia and COMISA groups, to achieve a more balanced population distribution, thus enhancing the robustness and generalizability of the findings reported here.

Several limitations refer to the diagnosis of insomnia and the information available in the dataset. Despite the criteria followed to establish the different groups aligned with previous research^24,25,29^, insomnia diagnosis was based on sleep questionnaires, implying that it was self-reported. Although it is a well-established method^6^, the presence of symptoms such as feeling unrested or having little energy is subject to non-objective impressions. Moreover, those questionnaires were not standardized tools specifically developed for diagnosing insomnia, and the SHHS dataset lacks information on chronic insomnia (i.e., insomnia persisting for over three months)^4^. Additionally, due to the intrinsic limitations of the database, information on diagnosed mental health conditions, such as depression or anxiety, was not available. Furthermore, the population under study has a high incidence of cardiovascular risks. Taken together, all these aspects could further influence HRV activity across the night. Therefore, while the findings reported in this study provide valuable insights, they should be interpreted with caution. Accordingly, the replication in future studies using databases specifically designed to evaluate COMISA, with the criteria based on more objective methods, such as actigraphy or medical evaluation^6^, while addressing the aforementioned limitations, remains essential to increase the generalization of the specific HRV alterations identified in the COMISA population.

Also related to insomnia diagnosis, in the present work, we have combined patients presenting any of the insomnia subtypes (onset insomnia, maintenance insomnia, and early morning awakening insomnia)^39^. Thus, despite the ANS dysfunction reported in this study in the Insomnia and COMISA populations, the evaluation of specific ANS dysfunction for each insomnia subtype, and its combination with OSA in the COMISA group, should be considered as a further step in future studies.

The analysis conducted in this study has been limited to characterizing HRV alterations over a single overnight ECG recording. Previous studies have demonstrated changes in HRV measures following the application of gold-standard treatments for insomnia^52,53^ and OSA^19,41^, consisting on cognitive behavioral therapy and continuous positive airway pressure, respectively. However, in the original SHHS study, despite including a follow-up group, no treatments for insomnia or OSA were administered, thus preventing the evaluation of treatment effects using this dataset. Consequently, assessing how treatments for both insomnia and OSA would impact the specific HRV alterations identified in COMISA patients represents a promising future research direction.

Finally, in the presence of respiratory efforts with the glottis closed, such as during the apneic events, the physiological interpretation of frequency ranges such as HF and LF classic frequency bands is hampered. Therefore, although the analysis of these frequency bands in the context of sleep apnea has been extensively performed, caution is needed when interpreting the results reached.

## Conclusion

This is the first study to confirm the presence of specific nocturnal ANS alterations due to the coexistence of insomnia and OSA in the same patients, characterized through time and frequency HRV analysis across the night and differentiating between wakefulness and sleep states. Concretely, it has been demonstrated that COMISA-specific ANS dysfunction primarily manifests as reduced PNS activity during wakefulness, evidenced by *RMSSD* reductions, along with heightened SNS excitation during sleep, indicated by increased *mHR* and *RP*_*BWOSA*_. This dysfunction also leads to decreases in *RP*_*BWMS*_ and *RP*_*BWCOMISA*_, a novel COMISA-specific feature introduced here. While the primary ANS dysfunction in COMISA patients appears due to the recurrence of apneic events during sleep, better captured through frequency analysis, insomnia significantly impacts their nocturnal HRV activity in the temporal domain. These findings underscore the importance of evaluating these HRV features in patients undergoing sleep studies, motivating further research in COMISA patients to gain insights and mitigate cardiovascular problems associated with this comorbid condition.

## Electronic supplementary material

Below is the link to the electronic supplementary material.

Supplementary Material 1

## Data Availability

The SHHS data is publicly available under request at https://sleepdata.org/datasets/shhs.
