# Supplementary material for: Heart rate variability analysis in comorbid insomnia and sleep apnea (COMISA)
Source: Sci Rep. 2025 May 21;15:17574. doi: 10.1038/s41598-025-02541-7 (PMC12095586; doi:10.1038/s41598-025-02541-7)
Supplement: Supplementary file 1 — Supplementary Material 1 [file 41598_2025_2541_MOESM1_ESM.docx]

**Heart rate variability analysis in comorbid insomnia and sleep apnea (COMISA)**

**Adrián Martín-Montero^a,b^, Fernando Vaquerizo-Villar^a,b^, Clara García-Vicente^a,b^*, Gonzalo C. Gutiérrez-Tobal^a,b^, Thomas Penzel^c^, and Roberto Hornero^a,b^**

^a^ Biomedical Engineering Group, University of Valladolid, Valladolid, Spain.

^b^ CIBER-BBN, Centro de Investigación Biomédica en Red en Bioingeniería, Biomateriales y Nanomedicina, Valladolid, Spain.

^c^ Charité-Universitätsmedizin, Interdisciplinary Center of Sleep Medicine, Berlin, Germany.

*** Corresponding author: Clara García-Vicente**

Biomedical Engineering Group, Facultad de Medicina, Av. Ramón y Cajal, 7, 47003 – Valladolid, Spain.

Tel. +34 983 423000 ext. 4713

E-mail address: [clara.garciav@uva.es](mailto:clara.garciav@uva.es)

URL: [www.gib.tel.uva.es](http://www.gib.tel.uva.es)

**Supplemental Information**

# Permutation tests to evaluate the influence of population imbalances

Following the application of the diagnostic criteria detailed in the Methods section to define Insomnia, OSA, and COMISA, the population distribution was as follows: 147 subjects in the COMISA group, 190 subjects in the Insomnia group, 2,260 subjects in the OSA group, and 2,738 subjects classified as No-OSA. This distribution presents a clear imbalance between groups, which may lead to statistically significant differences driven by the unequal sample sizes rather than actual variations in HRV behavior.

To deal with this, we implemented a strategy that combines random resampling with permutation tests for each comparison where a significant imbalance was present. Accordingly, the procedure was applied for all the features included in the study for the following group comparisons: No-OSA vs Insomnia, OSA vs Insomnia, No-OSA vs COMISA, and OSA vs COMISA. The application of permutation tests allows us to evaluate whether the observed differences in a selected metric of interest between groups exceed what would be expected under the null hypothesis, without requiring assumptions about the data distribution ^1,2^. By combining this approach with a random resampling procedure, we generated multiple balanced samples and assessed the stability of our results by constructing confidence intervals and evaluating statistical significance based on percentiles. The specific procedure followed for each comparison is detailed below.

Definition of the null hypothesis

To determine whether a true difference exists between groups for all features included in the study where an imbalance between populations is present, we defined the null hypothesis (*H_0_*) as: there is no real difference between groups and the observed difference occurred by chance. Accordingly, the alternative hypothesis (*H_A_*) was that a true difference exists between groups in the metric of interest. We selected the median of the features under study as the metric of interest.

Permutation test with random resampling procedure ^1,2^

To facilitate comprehension, we illustrate the procedure using a specific example: the differences in *mHR* during the wake-night (WN) period between the COMISA (n=147) and OSA (n=2,260) groups. The procedure is as follows ^1,2^:

1. Computation of the observed difference (*Dobs*):

$Dobs =\bar{mHR}_{OSA}- \bar{mHR}_{COMISA}$ (1)

where $\bar{mHR}$ represents the median *mHR* in the original sample for each group.

1. Extraction of a random subsample:

A subsample of *n* = 147 subjects is randomly selected from the original OSA sample.

1. Creation of permuted populations:

The original COMISA sample is combined with the OSA subsample extracted in step 2, and subjects are randomly assigned to create two new subsamples of equal size (n = 147 in this example), forming the permuted COMISA (Perm_COMISA) and permuted OSA (Perm_OSA) populations.

1. Computation of the permuted difference:

The difference in the metric of interest is calculated for the newly generated populations:

${Dperm}_{i}= \bar{mHR}_{Perm\_OSA}- \bar{mHR}_{Perm\_COMISA}$ (2)

1. Comparison with the observed difference:

At this point, we assess whether the absolute value of the permuted difference is greater than the observed difference and store the result:

$D_{i}=\left\{ \begin{aligned} 1 if \left| {Dperm}_{i} \right|\geq\left| Dobs \right| \\ 0 otherwise \end{aligned} \right.$ (3)

where *i* represents the *i*-th iteration of the permutation test. If $Dobs$ reflects a true difference, it is expected to be larger than the difference generated by randomly mixing both populations ^1,2^.

1. Repetition of the permutation process:

Steps 2-5 are repeated for N iterations, which we have fixed here as N=10,000, to construct a null distribution for hypothesis testing.

1. Evaluation of statistical significance:

The *p*-value can be computed as:

$p-value= \frac{\sum_{i=1}^{N} D_{i}}{N}$ (4)

If *p*-value < 0.01, we conclude, with a 99% confidence level, that a statistically significant difference exists, not driven by the imbalance between groups, and reject *H_0_*.

Accordingly, we performed the permutation test procedure to assess whether the imbalance between groups influenced the statistical significance of the differences observed in the HRV features computed in the study, restricting the analysis to comparisons with a considerable imbalance. The results obtained after permutation testing and applying FDR correction are shown in Table S1. Comparisons that did not reach statistical significance in this analysis cannot be considered true differences, even if they were initially deemed statistically significant. These differences are indicated in Table 2 in the main manuscript.

**Table S1.** Resulting *p*-values after performing permutation tests with random resampling to evaluate differences in HRV features between each pair of groups where there exists imbalance between populations.

| **Differences in HRV features computed across whole night** | | | | | | |
| --- | --- | --- | --- | --- | --- | --- |
| **Feature** | **No-OSA**  **vs**  **Insomnia** | **No-OSA**  **vs**  **OSA** | **No-OSA**  **vs**  **COMISA** | **Insomnia**  **vs**  **OSA** | **Insomnia**  **vs**  **COMISA** | **OSA**  **vs**  **COMISA** |
| ***mHR*** | **< 0.01** | N.A. | **< 0.01** | n.s. | N.A. | n.s. |
| ***SDNNI*** | n.s. | N.A. | n.s. | **< 0.01** | N.A. | n.s. |
| ***RMSSD*** | n.s. | N.A. | n.s. | n.s. | N.A. | n.s. |
| ***pNN50*** | n.s. | N.A. | n.s. | n.s. | N.A. | n.s. |
| ***RP_VLF_*** | n.s. | N.A. | n.s. | n.s. | N.A. | n.s. |
| ***RP_LF_*** | n.s. | N.A. | n.s. | n.s. | N.A. | n.s. |
| ***RP_HF_*** | n.s. | N.A. | n.s. | n.s. | N.A. | n.s. |
| ***LFn*** | n.s. | N.A. | n.s. | n.s. | N.A. | n.s. |
| ***RP_BWMS_*** | n.s. | N.A. | **< 0.01** | **< 0.01** | N.A. | n.s. |
| ***RP_BWOSA_*** | n.s. | N.A. | **< 0.01** | **< 0.01** | N.A. | n.s. |
| ***RP_BWCOMISA_*** | n.s. | N.A. | **< 0.01** | n.s. | N.A. | n.s. |
| ***RP_BWRes_*** | n.s. | N.A. | n.s. | n.s. | N.A. | n.s. |
| **Differences in HRV features computed across wake periods** | | | | | | |
| **Feature** | **No-OSA**  **vs**  **Insomnia** | **No-OSA**  **vs**  **OSA** | **No-OSA**  **vs**  **COMISA** | **Insomnia**  **vs**  **OSA** | **Insomnia**  **vs**  **COMISA** | **OSA**  **vs**  **COMISA** |
| ***mHR*** | n.s. | N.A. | n.s. | n.s. | N.A. | n.s. |
| ***SDNNI*** | n.s. | N.A. | **< 0.01** | n.s. | N.A. | **< 0.01** |
| ***RMSSD*** | n.s. | N.A. | **< 0.01** | n.s. | N.A. | n.s. |
| ***pNN50*** | n.s. | N.A. | n.s. | n.s. | N.A. | n.s. |
| ***RP_VLF_*** | n.s. | N.A. | n.s. | n.s. | N.A. | n.s. |
| ***RP_LF_*** | n.s. | N.A. | n.s. | n.s. | N.A. | n.s. |
| ***RP_HF_*** | n.s. | N.A. | n.s. | n.s. | N.A. | n.s. |
| ***LFn*** | n.s. | N.A. | n.s. | n.s. | N.A. | n.s. |
| ***RP_BWMS_*** | n.s. | N.A. | n.s. | n.s. | N.A. | n.s. |
| ***RP_BWOSA_*** | n.s. | N.A. | n.s. | n.s. | N.A. | n.s. |
| ***RP_BWCOMISA_*** | n.s. | N.A. | n.s. | n.s. | N.A. | n.s. |
| ***RP_BWRes_*** | n.s. | N.A. | n.s. | n.s. | N.A. | n.s. |
| **Differences in HRV features computed across sleep periods** | | | | | | |
| **Feature** | **No-OSA**  **vs**  **Insomnia** | **No-OSA**  **vs**  **OSA** | **No-OSA**  **vs**  **COMISA** | **Insomnia**  **vs**  **OSA** | **Insomnia**  **vs**  **COMISA** | **OSA**  **vs**  **COMISA** |
| ***mHR*** | **< 0.01** | N.A. | **< 0.01** | n.s. | N.A. | **< 0.01** |
| ***SDNNI*** | **< 0.01** | N.A. | n.s. | **< 0.01** | N.A. | n.s. |
| ***RMSSD*** | n.s. | N.A. | n.s. | n.s. | N.A. | n.s. |
| ***pNN50*** | n.s. | N.A. | n.s. | n.s. | N.A. | n.s. |
| ***RP_VLF_*** | n.s. | N.A. | n.s. | n.s. | N.A. | n.s. |
| ***RP_LF_*** | n.s. | N.A. | n.s. | n.s. | N.A. | n.s. |
| ***RP_HF_*** | n.s. | N.A. | n.s. | n.s. | N.A. | n.s. |
| ***LFn*** | n.s. | N.A. | n.s. | n.s. | N.A. | n.s. |
| ***RP_BWMS_*** | n.s. | N.A. | **< 0.01** | **< 0.01** | N.A. | n.s. |
| ***RP_BWOSA_*** | n.s. | N.A. | **< 0.01** | **< 0.01** | N.A. | n.s. |
| ***RP_BWCOMISA_*** | n.s. | N.A. | **< 0.01** | n.s. | N.A. | n.s. |
| ***RP_BWRes_*** | n.s. | N.A. | n.s. | n.s. | N.A. | n.s. |

n.s.: non-significant (*p*-value > 0.01).

Statistically significant comparisons (*p*-value < 0.01 after FDR correction) appear in bold.

N.A.: Not applicable (number of subjects included in the populations in the same order).

# HRV characterization across sleep stages

To further extend our characterization of nocturnal ANS alterations in the context of COMISA, we have performed the same analysis as in the WN, wake and sleep periods reported in the main manuscript, but splitting the sleep period into periods labeled as NREM and REM. To this end and based on the sleep stages annotations available in the database, we extracted the temporal and frequency domain features, evaluated statistically significant differences between groups, and conducted the permutation test as explained in the previous section. Table S2 shows the statistical significance (*p*-value < 0.01) obtained from comparisons between NREM and REM periods after applying FDR correction and surpassing the corresponding permutation test. For comparison purposes, the results across the sleep period have also been replicated in the table. Additionally, to further illustrate the differences, Figures S1 and S2 depict the boxplot distributions of the HRV features that reached any statistically significant differences across NREM and REM periods.

From Table S2 and Figures S1 and S2, it can be appreciated that several differences observed during the sleep period are present in both NREM and REM sleep stages and follow the same directional effect. For example, the increased activity in RPBWOSA in the OSA and COMISA groups, which differentiates these groups from the No-OSA and Insomnia populations, occurs in both sleep stages, with HRV activity increased in both cases. Therefore, by combining NREM and REM in the original approach, we reinforced the observed differences in mHR, SDNNI, RPHF, RPBWMS, RPBWOSA, and RPBWRES. Among all the differences, the most pronounced ones were again driven by OSA effects, when comparing HRV activity in RPBWMS and RPBWOSA from the No-OSA and Insomnia groups against OSA and COMISA.

**Table S2.** Statistically significant differences derived from the Mann-Whitney *U*-test in HRV features between each pair of groups considered in the sleep, NREM and REM periods, following FDR correction and permutation tests.

| **Differences in HRV features computed across sleep periods** | | | | | | |
| --- | --- | --- | --- | --- | --- | --- |
| **Feature** | **No-OSA**  **vs**  **Insomnia** | **No-OSA**  **vs**  **OSA** | **No-OSA**  **vs**  **COMISA** | **Insomnia**  **vs**  **OSA** | **Insomnia**  **vs**  **COMISA** | **OSA**  **vs**  **COMISA** |
| ***mHR*** | **< 0.01** | n.s. | **< 0.01** | n.s.* | n.s. | **< 0.01** |
| ***SDNNI*** | **< 0.01** | **< 0.01** | n.s. | **< 0.01** | n.s. | n.s. |
| ***RMSSD*** | n.s. | n.s. | n.s. | n.s. | n.s. | n.s. |
| ***pNN50*** | n.s. | n.s. | n.s. | n.s.* | n.s. | n.s. |
| ***RP_VLF_*** | n.s. | n.s. | n.s. | n.s. | n.s. | n.s. |
| ***RP_LF_*** | n.s. | n.s. | n.s. | n.s.* | n.s. | n.s. |
| ***RP_HF_*** | n.s. | **< 0.01** | n.s. | n.s. | n.s. | n.s. |
| ***LFn*** | n.s. | **< 0.01** | n.s. | n.s. | n.s. | n.s. |
| ***RP_BWMS_*** | n.s. | **< 0.01** | **< 0.01** | **< 0.01** | **< 0.01** | n.s. |
| ***RP_BWOSA_*** | n.s. | **< 0.01** | **< 0.01** | **< 0.01** | **< 0.01** | n.s. |
| ***RP_BWCOMISA_*** | n.s. | n.s. | **< 0.01** | n.s. | n.s. | n.s. |
| ***RP_BWRes_*** | n.s. | **< 0.01** | n.s. | n.s. | n.s. | n.s. |
| **Differences in HRV features computed across NREM periods** | | | | | | |
| **Feature** | **No-OSA**  **vs**  **Insomnia** | **No-OSA**  **vs**  **OSA** | **No-OSA**  **vs**  **COMISA** | **Insomnia**  **vs**  **OSA** | **Insomnia**  **vs**  **COMISA** | **OSA**  **vs**  **COMISA** |
| ***mHR*** | **< 0.01** | n.s. | **< 0.01** | n.s.* | n.s. | **< 0.01** |
| ***SDNNI*** | **< 0.01** | **< 0.01** | n.s. | **< 0.01** | n.s. | n.s. |
| ***RMSSD*** | n.s. | n.s. | n.s. | n.s.* | n.s. | n.s. |
| ***pNN50*** | n.s. | n.s. | n.s. | n.s.* | n.s. | n.s. |
| ***RP_VLF_*** | n.s. | n.s. | n.s. | n.s.* | n.s. | n.s. |
| ***RP_LF_*** | n.s. | **< 0.01** | n.s. | n.s.* | n.s. | n.s. |
| ***RP_HF_*** | n.s. | **< 0.01** | n.s. | n.s. | n.s. | n.s. |
| ***LFn*** | n.s. | **< 0.01** | n.s. | n.s.* | n.s. | n.s. |
| ***RP_BWMS_*** | n.s. | **< 0.01** | **< 0.01** | **< 0.01** | **< 0.01** | n.s. |
| ***RP_BWOSA_*** | n.s. | **< 0.01** | **< 0.01** | **< 0.01** | **< 0.01** | n.s. |
| ***RP_BWCOMISA_*** | n.s. | n.s. | **< 0.01** | n.s. | n.s. | n.s. |
| ***RP_BWRes_*** | n.s. | **< 0.01** | n.s. | n.s. | n.s. | n.s. |
| **Differences in HRV features computed across REM periods** | | | | | | |
| **Feature** | **No-OSA**  **vs**  **Insomnia** | **No-OSA**  **vs**  **OSA** | **No-OSA**  **vs**  **COMISA** | **Insomnia**  **vs**  **OSA** | **Insomnia**  **vs**  **COMISA** | **OSA**  **vs**  **COMISA** |
| ***mHR*** | **< 0.01** | n.s. | **< 0.01** | n.s.* | n.s. | **< 0.01** |
| ***SDNNI*** | **< 0.01** | **< 0.01** | n.s. | **< 0.01** | n.s. | n.s. |
| ***RMSSD*** | n.s. | n.s. | n.s. | n.s. | n.s. | n.s. |
| ***pNN50*** | n.s. | n.s. | n.s. | n.s. | n.s. | n.s. |
| ***RP_VLF_*** | n.s. | **< 0.01** | n.s.* | n.s. | n.s. | n.s. |
| ***RP_LF_*** | n.s. | **< 0.01** | n.s.* | n.s. | n.s. | n.s. |
| ***RP_HF_*** | n.s. | **< 0.01** | n.s.* | n.s. | n.s. | n.s. |
| ***LFn*** | n.s. | n.s. | n.s. | n.s. | n.s. | n.s. |
| ***RP_BWMS_*** | n.s. | **< 0.01** | **< 0.01** | **< 0.01** | **< 0.01** | n.s. |
| ***RP_BWOSA_*** | n.s. | **< 0.01** | **< 0.01** | **< 0.01** | **< 0.01** | n.s. |
| ***RP_BWCOMISA_*** | n.s. | **< 0.01** | **< 0.01** | n.s. | n.s. | n.s. |
| ***RP_BWRes_*** | n.s. | **< 0.01** | n.s.* | n.s. | n.s. | n.s. |

n.s.: non-significant (*p*-value > 0.01).

*deemed as non-significant after permutation tests.

Statistically significant comparisons (*p*-value < 0.01 after FDR correction) appear in bold


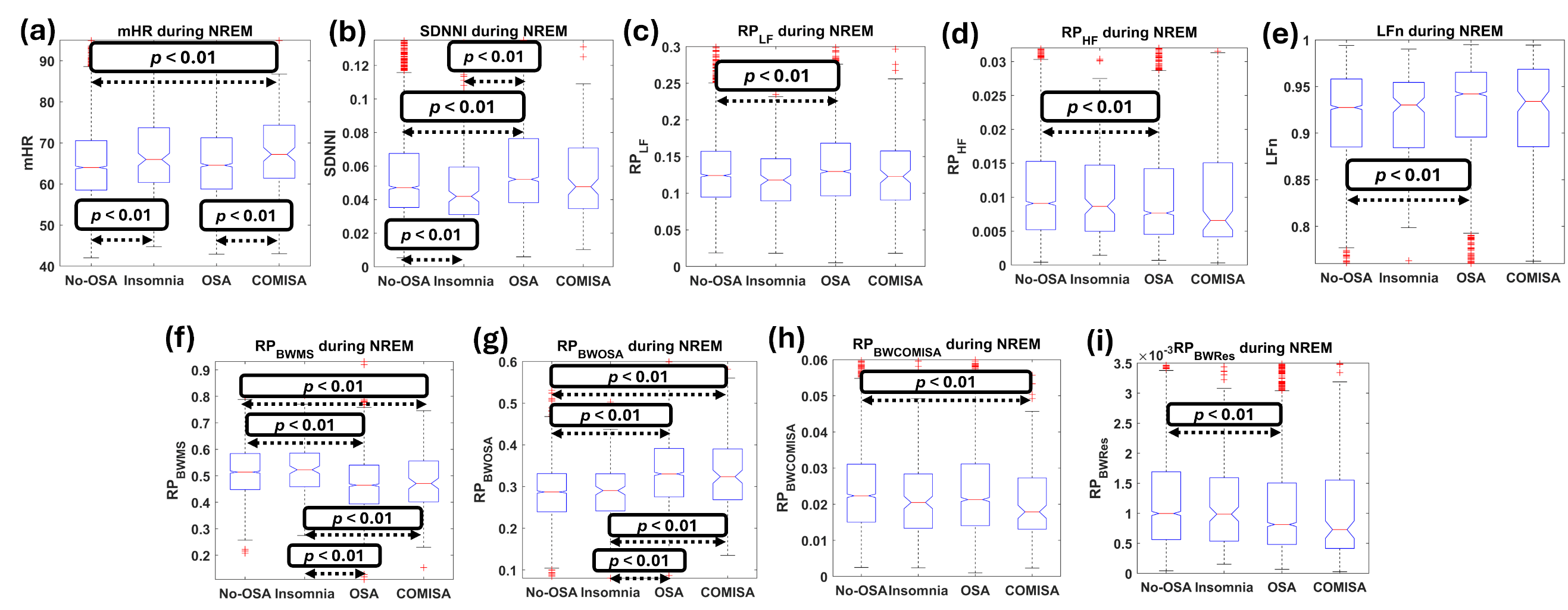


**Figure S1.** Boxplot distribution of the features computed across NREM periods that reached statistically significant differences between any group comparisons. Statistically significant differences are highlighted within each corresponding subplot, with arrows delimiting where the differences arise. (a) *mHR* boxplots; (b) *SDNNI* boxplots; (c) *RP_LF_* boxplots; (d) *RP_HF_* boxplots; (e) *LFn* boxplots; (f) *RP_BWMS_* boxplots; (g) *RP_BWOSA_* boxplots; (h) *RP_BWCOMISA_* boxplots; (i) *RP_BWRes_* boxplots.


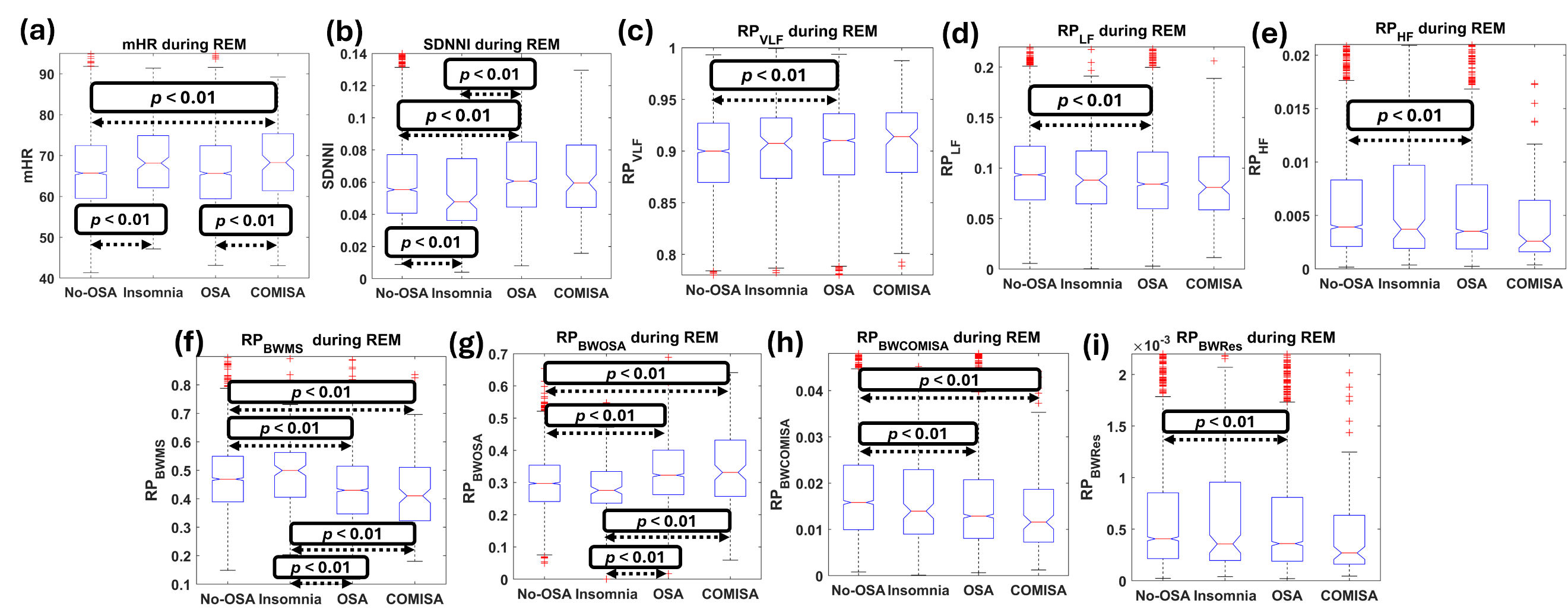


**Figure S2.** Boxplot distribution of the features computed across REM periods that reached statistically significant differences between any group comparisons. Statistically significant differences are highlighted within each corresponding subplot, with arrows delimiting where the differences arise. (a) *mHR* boxplots; (b) *SDNNI* boxplots; (c) *RP_VlF_* boxplots; (d) *RP_LF_* boxplots; (e) *RP_HF_* boxplots; (f) *RP_BWMS_* boxplots; (g) *RP_BWOSA_* boxplots; (h) *RP_BWCOMISA_* boxplots; (i) *RP_BWRes_* boxplots.

It is also noteworthy that *RP_LF_* reached statistically significant differences between the No-OSA and OSA groups in both NREM and REM sleep stages, as did *RP_VLF_*, but only during REM. However, when analyzing the entire sleep period, these differences did not appear. This can be attributed to the opposing trends observed in HRV activity within these frequency ranges during NREM and REM, as illustrated for *RP_LF_* (see Figures S1c and S2d). Consequently, these opposing effects mask the overall differences between the No-OSA and OSA groups when considering the complete sleep period. Additionally, the influence of *RP_LF_* on *LFn* partially masks the differences between these two groups during REM. However, this effect seems insufficient to obscure the differences across the entire sleep period, as the consistent behavior of *RP_HF_* during sleep, which also contributes to *LFn*, helps to maintain statistical significance. The original motivation for identifying spectral bands of interest in the context of OSA was to propose alternatives to the conventional HRV frequency ranges ^3^. Specifically, BWMS was introduced as an alternative to VLF for evaluating OSA effects, as it falls within the VLF range, while BWOSA was proposed as an alternative to LF, which had traditionally been used to assess sympathetic activation in OSA ^4,5^. The consistent differences observed here across sleep stages between the No-OSA and OSA groups in *RP_BWMS_* and *RP_BWOSA_*, but not in *RP_VLF_* and *RP_LF_*, highlight the value of these newly defined frequency ranges in assessing OSA-related effects in adults, as opposed to the classical spectral frequency bands, which seem to mask apneic effects.

Finally, the most relevant findings obtained from analyzing HRV behavior between NREM and REM sleep stages emerge when evaluating the novel BWCOMISA frequency range. By analyzing BWCOMISA activity during the complete sleep period, statistically significant differences were observed only between the No-OSA and COMISA groups, with an activity reduction in the latter. Similarly, by splitting the sleep periods between NREM and REM sleep stages, the observed differences between No-OSA and COMISA groups remained in both stages. However, an additional statistically significant difference appeared during REM sleep between the No-OSA and OSA groups. The frequency range covered by LF (0.04-0.15 Hz) is a priori associated with SNS and PNS activation, whereby parasympathetic activation has a rapid response time, and sympathetic activation occurs more slowly ^6^. Accordingly, it can be stated that BWCOMISA (0.071 to 0.11 Hz) reflects part of the periodic PNS modulation of HRV. It is known that during NREM, there is an intrinsic basal PNS activation ^7,8^. This could explain why No-OSA and OSA groups did not reach statistically significant differences in BWCOMISA activity, as OSA patients seem to retain the basal PNS function across this frequency range. In contrast, COMISA patients exhibit persistently reduced PNS activity even during NREM, suggesting a more profound autonomic dysfunction (see Figure S1h). From Figure S2h, it can be appreciated that BWCOMISA activity is significantly reduced in both OSA and COMISA patients during REM sleep, indicating impaired PNS function in both groups. However, as shown in Table 1 in the main manuscript, the time spent in NREM is considerably longer than the time spent in REM. As a result, when analyzing the complete sleep period, the cumulative effect of PNS impairment captured by BWCOMISA becomes statistically significant only in the COMISA group, distinguishing it as a specific HRV characteristic of this population.

**SUPPLEMENTAL REFERENCES**

1. Good, P. *Permutation Tests: A Practical Guide to Resampling Methods for Testing Hypotheses*. (Springer New York, 2000). doi:10.1007/978-1-4757-3235-1.

2. Pesarin, F. & Salmaso, L. *Permutation Tests for Complex Data: Theory, Applications and Software*. (Wiley, 2010). doi:10.1002/9780470689516.

3. Martín-Montero, A. *et al.* Heart rate variability spectrum characteristics in children with sleep apnea. *Pediatr. Res.* **89**, 1771–1779 (2021).

4. Vanninen, E., Tuunainen, A., Kansanen, M., Uusitupa, M. & Länsimies, E. Cardiac sympathovagal balance during sleep apnea episodes. *Clin. Physiol.* **16**, 209–216 (1996).

5. Dingli, K. *et al.* Spectral oscillations of RR intervals in sleep apnoea/hypopnoea syndrome patients. *Eur. Respir. J.* **22**, 943–950 (2003).

6. Nisbet, L. C., Yiallourou, S. R., Walter, L. M. & Horne, R. S. C. Blood pressure regulation, autonomic control and sleep disordered breathing in children. *Sleep Med. Rev.* **18**, 179–189 (2014).

7. Cabiddu, R., Cerutti, S., Viardot, G., Werner, S. & Bianchi, A. M. Modulation of the Sympatho-Vagal Balance during Sleep: Frequency Domain Study of Heart Rate Variability and Respiration. *Front. Physiol.* **3**, (2012).

8. Trinder, J. *et al.* Autonomic activity during human sleep as a function of time and sleep stage. *J. Sleep Res.* **10**, 253–264 (2001).
